# Inclusion complexes of β-cyclodextrin with tricyclic drugs: an X-ray diffraction, NMR and molecular dynamics study

**DOI:** 10.3762/bjoc.13.70

**Published:** 2017-04-13

**Authors:** Franca Castiglione, Fabio Ganazzoli, Luciana Malpezzi, Andrea Mele, Walter Panzeri, Giuseppina Raffaini

**Affiliations:** 1 Dipartimento di Chimica, Materiali e Ingegneria Chimica ‘G. Natta’, Politecnico di Milano, via Mancinelli 7, 20131 Milano, Italy; 2 CNR-Istituto di Chimica del Riconoscimento Molecolare – Via Mancinelli 7, 20131 Milano, Italy

**Keywords:** amitriptyline, β-cyclodextrin, crystal structure, cyclobenzaprine, molecular dynamics simulations, NOE

## Abstract

Tricyclic fused-ring cyclobenzaprine (**1**) and amitriptyline (**2**) form 1:1 inclusion complexes with β-cyclodextrin (β-CD) in the solid state and in water solution. Rotating frame NOE experiments (ROESY) showed the same geometry of inclusion for both **1**/β-CD and **2**/β-CD complexes, with the aromatic ring system entering the cavity from the large rim of the cyclodextrin and the alkylammonium chain protruding out of the cavity and facing the secondary OH rim. These features matched those found in the molecular dynamics (MD) simulations in solution and in the solid state from single-crystal X-ray diffraction of **1**/β-CD and **2**/β-CD complexes. The latter complex was found in a single conformation in the solid state, whilst the MD simulations in explicit water reproduced the conformational transitions observed experimentally for the free molecule.

## Introduction

**Figure 1:**
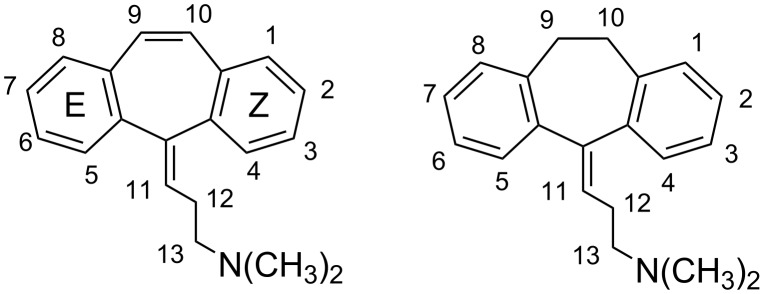
Molecular formulae and atom numbering of cyclobenzaprine (**1**, left) and amitriptyline (**2**, right). E and Z symbols are arbitrarily introduced to identify the two aromatic rings.

The present paper reports on a multidisciplinary approach [1–2] based on single crystal X-ray diffraction, solution NMR spectroscopy and molecular dynamics (MD) simulations with explicit water to study the inclusion complexes of two tricyclic aromatic molecules – cyclobenzaprine (**1**) and amitriptyline (**2**, Figure 1) – with β-cyclodextrin (β-CD). Previous work already considered certain aspects of the interaction of **2** with β-CD [3–8], but no full characterization of the complex geometry in solution and in the solid state was carried out, while the only similation study of this complex was very limited both in scope and in the adopted methodology [6]. In addition, to the best of our knowledge no study was ever performed on the inclusion complex of the strictly related compound **1**. Compounds **1** and **2** are not planar and the exocyclic double bond prevents the free rotation of the side chain with respect to the ring system. Consequently, **1** and **2** show inherent chirality [9] as lacking of symmetry elements. The main purpose of the work is the comparison of the structural features obtained in the solid state and in D_2_O solution by X-ray diffraction and NMR spectroscopy, respectively. The dynamic behaviour of the examined systems is simulated by MD runs of the complexes with explicit water. Suitable structural descriptors thus obtained as time averages are then compared to those found experimentally.

## Results and Discussion

**Figure 2:**
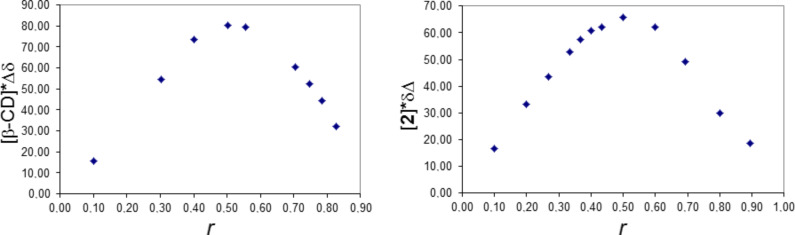
Left: Job’s plot for H3’ chemical shift variations of the complex β-CD/**1**. Right: Job’s plot for H11 (see Figure 1 for atom numbering) chemical shift variations of the complex β-CD/**2**.

The guest molecules **1** and **2** form 1:1 inclusion complexes with β-CD in aqueous solution and in the solid state. The Job’s plots are reported in Figure 2.

The plots of Figure 2 show that the maximum of the curves is obtained, in both cases, for *r* = 0.5, consistent with the 1:1 host–guest stoichiometry.

**Figure 3:**
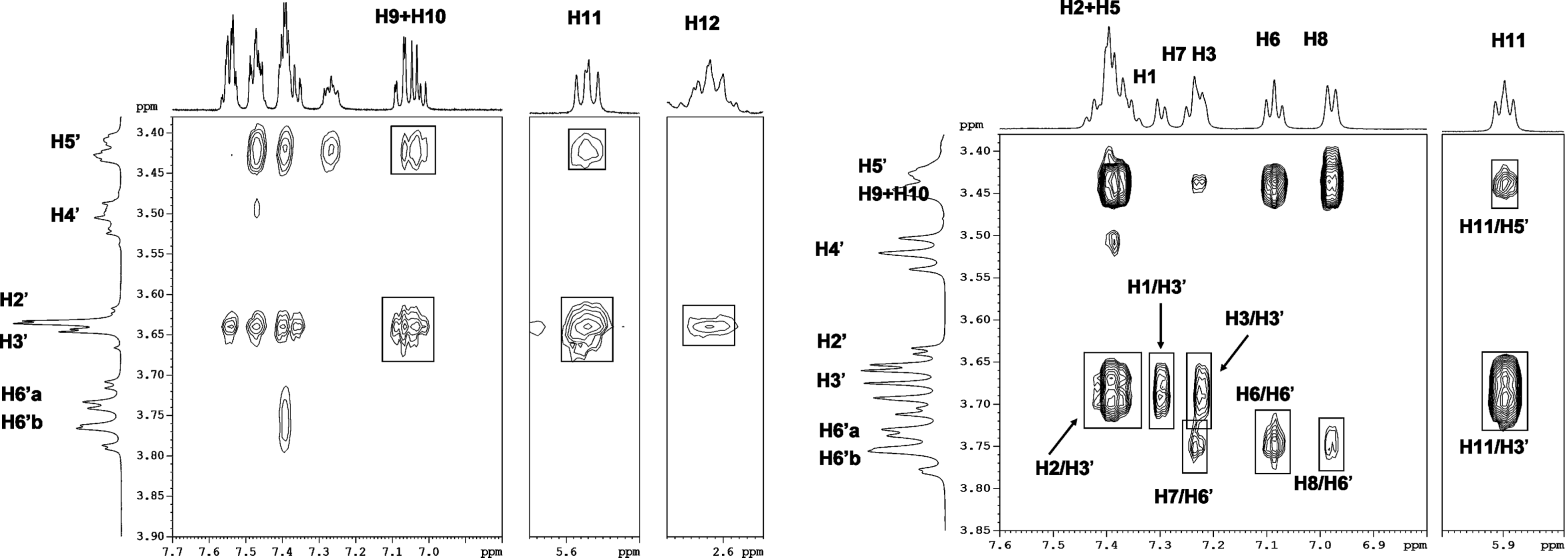
Expansion of 2D-ROESY of **1**/β-CD (left) and **2**/β-CD (right) complexes. Atom numbering is referred to Figure 1. Primed numbers are used for glucose units.

Some important structural features of the inclusion complexes of **1** and **2** can be outlined by the analysis of the ^1^H NMR spectra: the spectrum of **1** shows that the AB quartet assigned to H9–H10 spin system is split into two AB quartets on passing from pure **1** to the corresponding **1**/β-CD complex, thus showing the formation of two diastereomeric inclusion complexes. Similar behaviour can be reported for **2**. The geometry of inclusion can be inferred by analysis of intermolecular NOEs obtained from ROESY spectra (Figure 3).

**Figure 4:**
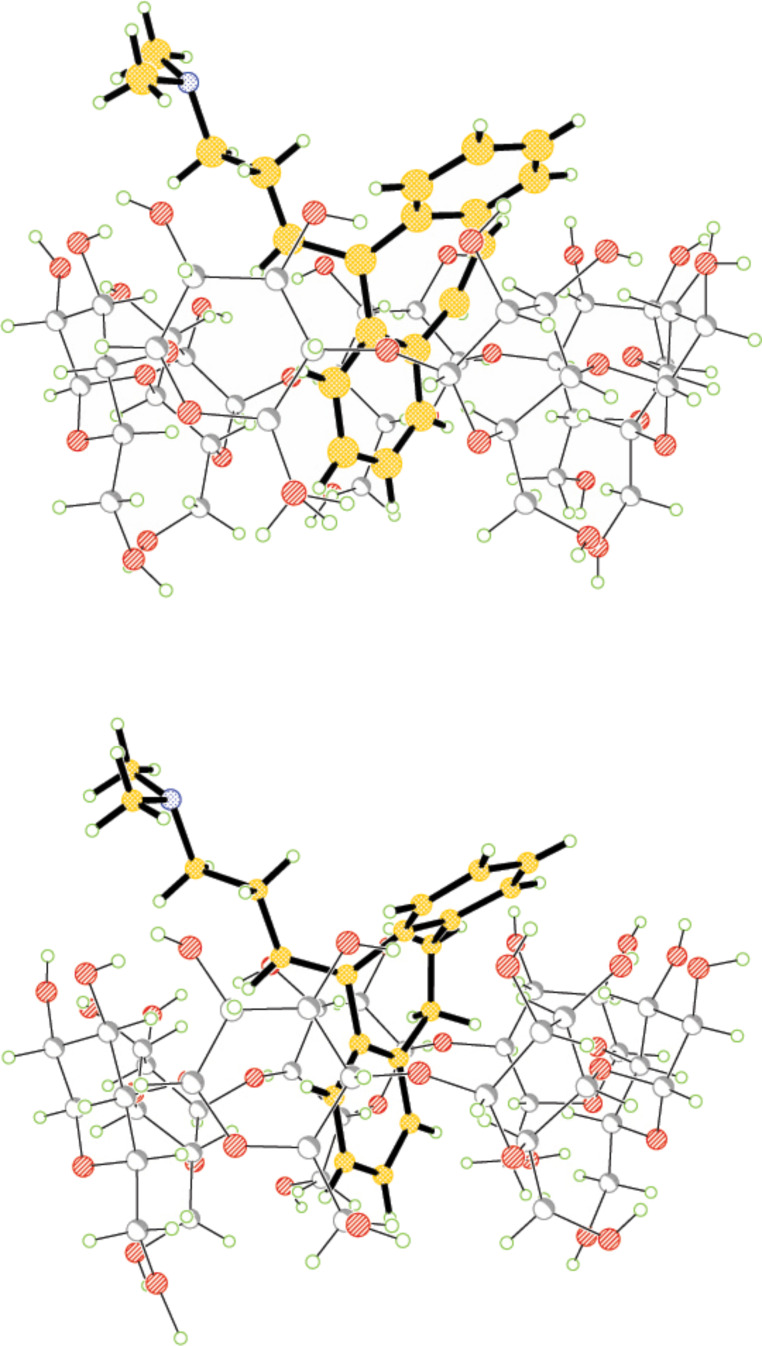
X-ray diffraction structures of **1**/β-CD (top) and **2**/β-CD (bottom) complexes.

The signals of H9, H10 and H11 of **1** show intermolecular contacts with H5’ and H3’ of the β-CD, indicating that the ring system of **1** is deeply inserted into the cavity of the β-CD. The selective NOE between H12 and H3’ suggests that the alkylammonium chain is protruding out of the β-CD cavity from the secondary OH rim. The analysis of intermolecular NOEs within the **2**/β-CD complex points out that the overall geometry is very much similar to that described for **1**/β-CD. The approximate and qualitative picture derived from NOE restraints is in good agreement with the solid state structure of the two complexes obtained from single crystal X-ray diffraction. The refined structures are shown in Figure 4.

As expected, the most significant difference between the two structures involves the C9–C10 bond. The C9–C10 bond distances for **1** and **2** are 1.312(14) Å and 1.420(8) Å, respectively, while the torsion angles around this bond are 0.6(19)° and 51.3(10)° for **1** and **2**, respectively, where the figures in parentheses give the standard errors.

The β-CD macrocyclic rings appear slightly distorted upon inclusion of the guest molecule. In the crystal, the arrangement of the complexes formed by **1** and **2** with β-CD are similar, being stacked head-to-head to form antiparallel columns along the crystallographic *b* axis. Within each column, the molecules are linked by hydrogen bonding involving both the macrocycles and the guest molecules, while interactions between macrocycles link adjacent columns. A large network of H-bonds involving the water molecules contribute to the crystal stability.

In both cases the crystal structures are non-centrosymmetric, indicating that the crystal contains a single enantiomer of **1** and **2**. The overall topology of inclusion matches that found in solution and through molecular simulations. In both cases the complex structures do not show any disorder: this finding is largely predictable for the rigid tricyclic moiety of **1** but it is remarkable in **2**. Indeed, literature data on isolated amitriptyline point out that the fused ring system of **2** shows conformational transitions [10], especially those involving the torsion about the C9–C10 single bond. The lack of disorder in the C9–C10 segment suggests that complexation constrains **2** in a single conformation in the solid state.

**Figure 5:**
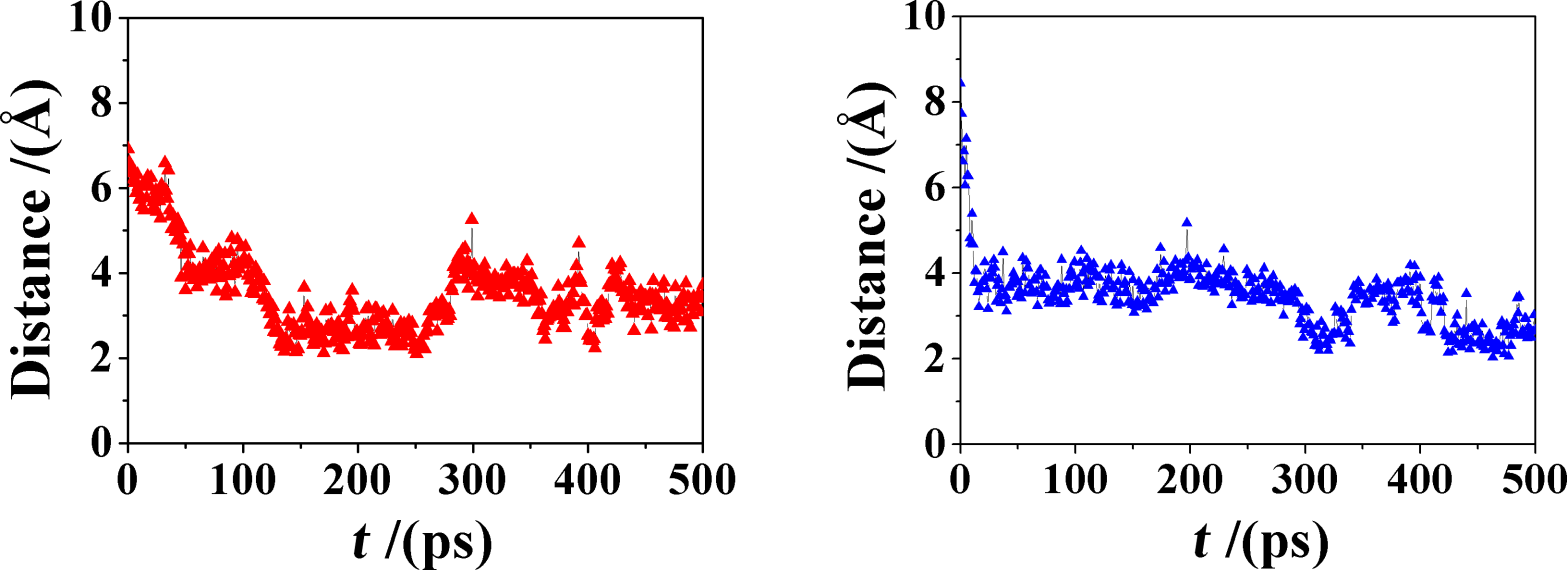
The distance between the center of mass (c.o.m.) of molecule **1** (at left) and of molecule **2** (at right) from the c.o.m. of the β-CD.

The MD simulations led to the formation of a 1:1 inclusion complex of β-CD with molecules **1** and **2** both in vacuo and in explicit water. In both cases, the complex formation was relatively fast, and allowed us to find the most stable geometry eventually achieved from the trial starting arrangements [17] mentioned in the Materials and Methods section. The most stable complex yielded inclusion of an aromatic ring in the β-CD, with the seven-membered ring, the side chain and the other aromatic ring protruding above the secondary rim, quite similar to the arrangement experimentally determined in the solid state by X-ray diffraction with a very similar depth of inclusion. In view of the geometrical similarity achieved in the two different simulation environments, in the following we will only discuss the results obtained for the simulations in explicit water. In water, the inclusion process was relatively fast, as it can be gauged by Figure 5, which shows the distance between the center of mass (c.o.m.) of molecule **1** and **2** and of the β-CD.

**Figure 6:**
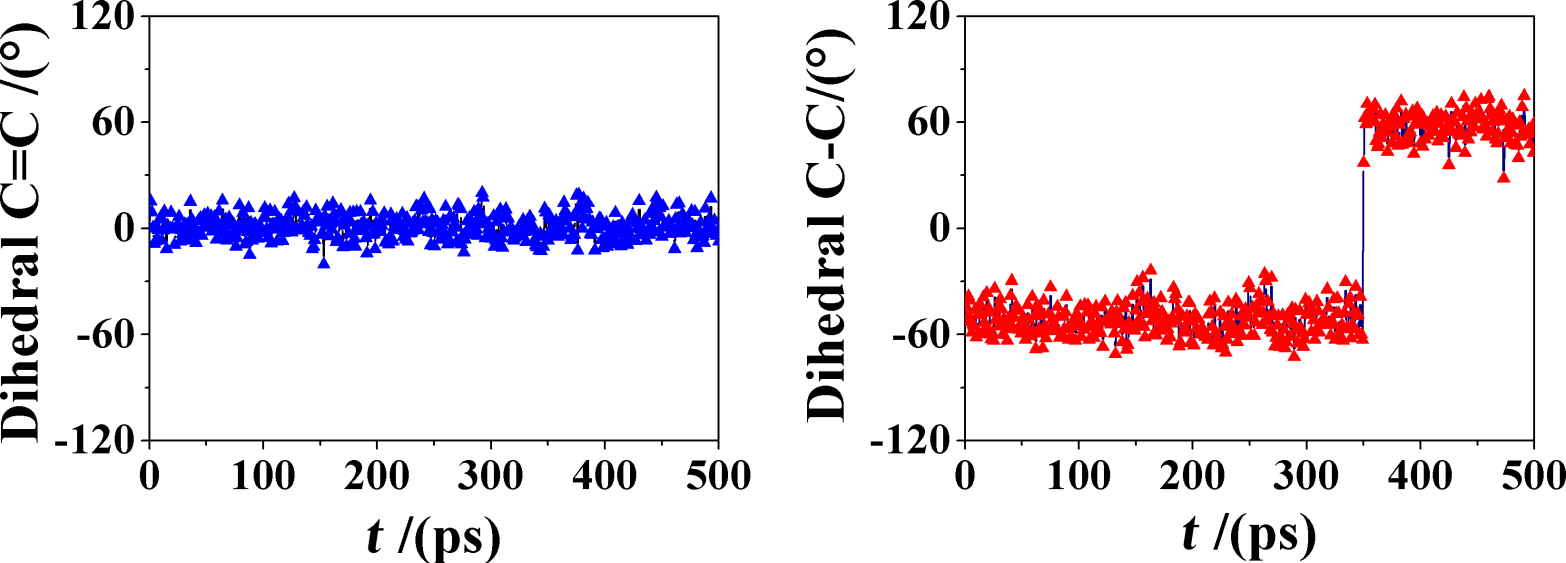
The value of the C9–C10 dihedral angle as a function of time for the complexes of molecule **1** and **2** (left and right, respectively).

**Figure 7:**
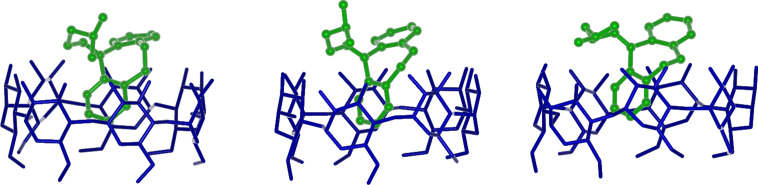
Snapshots of the conformational transition in **2**/β-CD in water taken at a 1 ps interval.

It is interesting to note that the inclusion is much faster in the case of molecule **2**, being essentially complete within the initial 20–30 ps of the MD run, apart from some smaller and lengthier rearrangements at longer times. Such very fast process is possibly related to the larger or smaller rigidity of the central ring in the tricyclic system: in fact, molecule **2** requires a smaller time interval before inclusion thanks to its larger fluctuations, related in turn to the flexibility of the central cycle, not constrained by the C9–C10 double bond present in molecule **1**. The larger flexibility of molecule **2** is best shown through the torsional degree of freedom around its C9–C10 bond that is experimentally observed in solution for uncomplexed **2** [10], and is also preserved in the included state, according to the present MD simulations in water. This torsional freedom is reported in Figure 6, where we show the value of the C9–C10 dihedral angle as a function of time for both complexes. It may be clearly seen that this dihedral angle fluctuates around a value of 0° in molecule **1**, being constrained by the double bond. Conversely, in molecule **2** it undergoes a sharp and very fast change from an average value of (−52.3(5) ± 8.9)° to a value of (+57.7(6) ± 8.0)°, where the value in parenthesis is the standard error on the last significant digit of the mean, and the ± sign indicates the standard deviation around the mean, indicating relatively large fluctuations. In this connection, we also note that this conformational transition between two gauche states is very fast, being completed within 3 ps only. Figure 7 shows three snapshots of the complex taken at a 1 ps interval during this conformational transition, showing the quite large rearrangement in water of the moiety protruding over the secondary rim. It is interesting to note that this change in the C9–C10 dihedral angle can only takes place when the thermal fluctuations of the complex lead to some small upward displacement of the guest molecule with respect to β-CD thus allowing for a larger conformational freedom of the central ring of molecule **2** not constrained by the secondary rim of the host.

## Conclusion

The integrated approach X-ray/NMR/MD was successfully applied to the structure assessment of **1**/β-CD and **2**/β-CD complexes. The crystallization of a single enantiomer of **1** encapsulated into β-CD showed that the latter acts as chiral selector towards racemic **1**. The comparison of the X-ray structure and the MD simulations in water of **2**/β-CD complex showed that **2** is present as single conformer in the crystal and in two conformations in the solution state.

## Materials and Methods

### X-ray diffraction

Single crystals of **1**/β-CD and **2**/β-CD were obtained after many attempts by slow evaporation of the solvent from an aqueous solution. The **1**/β-CD complex appeared extremely unstable in the air and finally a poor quality crystal, just enough suitable for the X-ray diffraction, was sealed in a glass capillary in the presence of the mother liquor. Data collection was performed on a Siemens P4 diffractometer using Cu Kα (λ = 1.54178 Å) radiation for **1** and on a Bruker SMART APEX II diffractometer equipped with APEX II CCD detector using Mo Kα (λ = 0.71073 Å) radiation for **2**. The structures were solved by direct methods with the SIR97 program [11] and refined by full-matrix-least-squares procedure with the SHELX97 program [12]. The refinement of **1**, owing to the low ratio of data/refined parameters, was refined by block full-matrix-least-squares procedure. All non H atoms of both complexes were refined anisotropically. The H atoms were positioned in the calculated positions and refined with a riding model. Both complexes were found to crystallize in the solid state in 1:1 host–guest ratio with many partially disordered water molecules distributed in the intermolecular space outside the macrocyclic cavities. Full molecular and crystal data, together with the structural refinement details, are given in Supporting Information File 1 (Tables S1–S14 and Figures S1–S4 for the complexed guest geometry and for the packing diagrams) and Supporting Information Files 2 and 3 (cif files).

### NMR spectroscopy

The NMR spectra were recorded in D_2_O on a Bruker Avance 500 spectrometer at 305 K. The stoichiometry of complexes was assessed by the Job’s method [13]. A typical procedure was as follows: (i) two stock solutions of host (β-CD) and guest (compound **1** or **2**) were prepared at given concentrations; (ii) accurately measured volumes of host and guest solutions were mixed in different volumes ratios in the NMR tubes and in such a way that the total volume was 750 μL for all the solutions; (iii) the NMR spectra were collected for each sample and the chemical shift variation Δδ were measured for some target proton signal; (iv) the data were used for the plot of [β-CD]*Δδ vs *r* or [guest]*Δδ vs *r* where *r* = [host]/[host]+[guest] (or related expression in the case the guest’s chemical shift variations are reported), providing the Job’s plot with the typical bell shape. The abscissa of the maximum provides the stoichiometry of the host–guest complex in solution. 2D NOE correlation experiments in the rotating frame (ROESY) were acquired on 4 mM solutions by using a suitable pulse sequence with two different transmitter offsets for spin-lock and pulse [14] in order to minimize artefacts due to the J-coupling magnetization transfer (HOHAHA). The typical experimental set-up was as follows: 2K points acquired in the F2 domain, 512 increments and subsequent zero-filling to 1K to process data.

### Molecular dynamics simulations

The simulations employed InsightII/Discover [15] with the CVFF force field [16]. The structure of molecules **1** and **2** were first subjected to an MD run in vacuo and finally optimized up to an energy gradient lower than 4 × 10^−3^ kJ mol^−1^ Å^−1^. The simulation protocol closely followed the strategy proposed by some of us for modeling the inclusion complex formation without any a priori assumption about its possible geometry [17–21]. Thus, the optimized molecules were placed close to β-CD in 12 unbiased trial geometries with the main sides close to the two rims and the outer surface of β-CD in different orientations: no inclusion complex was assumed at the beginning of the simulations. The simulations in water were carried out in a large cell with periodic boundary conditions. The outer adducts were separately optimized in vacuo and in explicit water adopting a box of water with a size of 33 Å adopting periodic boundary conditions, and then subjected to MD runs (2 ns in vacuo, 500 ps in water) at room temperature (300 K). The dynamic equations were integrated using the Verlet algorithm with a time step of 1 fs at a temperature of 300 K, controlled through the Berendsen thermostat. Equilibration of the resulting adducts was monitored through the time change of the total energy and of its components (including also the van der Waals components) and of the distance between the centers of mass of the host and of the guest molecule [17]. Final optimizations (up to an energy gradient lower than 4 × 10^−3^ kJ mol^−1^ Å^−1^) of many conformations generated during the MD runs yielded the most stable host–guest geometries discussed in the main text.

## Supporting Information

File 1 Crystallographic data for cyclobenzaprine (**1**) and amitriptyline (**2**).
Tables with the crystallographic data, the atomic coordinates, the bond distances and angles, the torsion angles and the hydrogen bonds (Tables S1–S14) and the geometry of the complexed guest and the the packing diagram in the crystalline state (Figures S1–S4).

File 2 Chemical information file for cyclobenzaprine (**1**).

File 3 Chemical information file for amitriptyline (**2**).
