# Supplementary material for: Inclusion complexes of β-cyclodextrin with tricyclic drugs: an X-ray diffraction, NMR and molecular dynamics study
Source: Beilstein J Org Chem. 2017 Apr 13;13:714–9. doi: 10.3762/bjoc.13.70 (PMC5405689; doi:10.3762/bjoc.13.70)
Supplement: File 1 — Crystallographic data for cyclobenzaprine (1) and amitriptyline (2). Tables with the crystallographic data, the atomic coordinates, the bond distances and angles, the torsion angles and the hydrogen bonds (Tables S1–S14) and the geometry of the complexed guest and the the packing diagram in the crystalline state (Figures S1–S4). [file Beilstein_J_Org_Chem-13-714-s001.pdf]

Supporting Information

for

Inclusion complexes of β-cyclodextrin with tricyclic drugs: an X-ray diffraction, NMR and molecular dynamics study

Franca Castiglione[1], Fabio Ganazzoli[1], Luciana Malpezzi*[1], Andrea Mele*[1,2], Walter Panzeri[2] and Giuseppina Raffaini*[1]

Address: [1]Dipartimento di Chimica, Materiali e Ingegneria Chimica 'G. Natta', Politecnico di Milano, via Mancinelli 7, 20131 Milano, Italy and [2]CNR-Istituto di Chimica del Riconoscimento Molecolare – Via Mancinelli 7, 20131 Milano, Italy

Email: Luciana Malpezzi - luciana.malpezzi@polimi.it; Andrea Mele - andrea.mele@polimi.it; Giuseppina Raffaini - giuseppina.raffaini@polimi.it

* Corresponding author

Crystallographic data for cyclobenzaprine (1) and amitryptiline (2)

Crystallographic data for cyclobenzaprine (**1**)

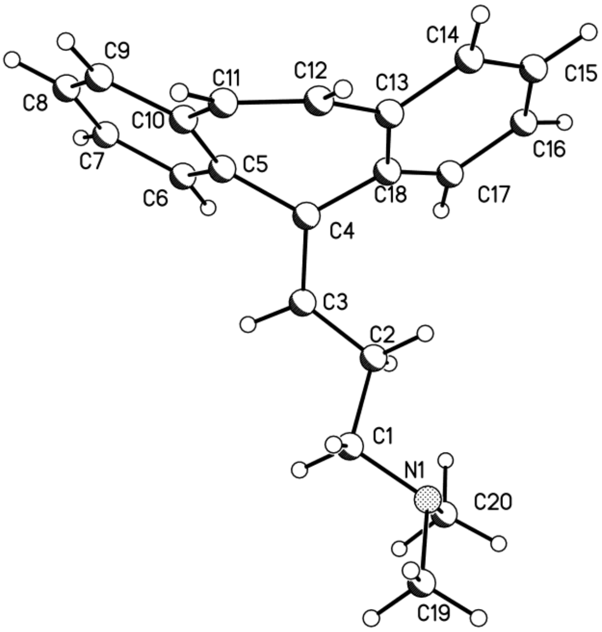

Figure S1: The structure of compound **1** in the inclusion complex with the atom numbering scheme from the X-ray results.

Table S1: Crystal data and structure refinement for cdbzp-16.

Identification code	cdbzp-16
Empirical formula	C62 H91 N O50.10
Formula weight	1651.96
Temperature	293(2) K
Wavelength	1.54178 Å
Crystal system, space group	orthorhombic, P212121
Unit cell dimensions	a = 15.361(12)Å alpha = 90 deg.
	b = 17.609(12)Å beta = 90 deg.
	c = 27.316(16)Å gamma = 90 deg.
Volume	7389(9) A^3
Z, Calculated density	4, 1.485 Mg/m^3
Absorption coefficient	1.139 mm^-1
F(000)	3483
Crystal size	0.25 x 0.15 x 0.08 mm
Theta range for data collection	6.61 to 87.64 deg.
Limiting indices	0<=h<=18, 0<=k<=22, 0<=l<=35
Reflections collected / unique	8189 / 8189 [R(int) = 0.0000]
Completeness to theta	87.64 89.3 %
Refinement method	Full-matrix-block least-squares on F^2
Data / restraints / parameters	8189 / 0 / 1087
Goodness-of-fit on F^2	1.046
Final R indices [I>2sigma(I)]	R1 = 0.0780, wR2 = 0.1952
R indices (all data)	R1 = 0.1473, wR2 = 0.2413
Absolute structure parameter	1.2(4)
Extinction coefficient	0.00021(12)
Largest diff. peak and hole	0.421 and -0.305 e.A^-3

Table S2: Atomic coordinates (x 10^4) and equivalent isotropic displacement parameters (A^2 x 10^3) for cdbzp-16. U(eq) is defined as one third of the trace of the orthogonalized Uij tensor.

	x	y	z	U(eq)
C(1A)	2520(5)	983(5)	7759(3)	38(2)
C(2A)	2584(5)	359(5)	8079(3)	41(2)
O(2A)	3036(4)	-217(4)	7854(2)	50(2)
C(3A)	1677(5)	127(4)	8248(3)	35(2)
O(3A)	1806(4)	-411(4)	8601(2)	53(2)
C(4A)	1167(5)	754(5)	8419(3)	38(2)
O(4A)	310(3)	504(3)	8547(2)	34(1)
C(5A)	1116(6)	1322(5)	8046(3)	41(2)
O(5A)	1998(4)	1542(3)	7938(2)	43(1)
C(6A)	590(8)	1983(5)	8174(4)	59(3)
O(6A)	875(6)	2290(5)	8596(3)	84(3)
C(1B)	2555(5)	940(5)	5981(3)	36(2)
C(2B)	2912(6)	275(5)	6205(3)	42(2)
O(2B)	2712(4)	-349(4)	5935(2)	54(2)
C(3B)	2501(5)	185(4)	6663(3)	32(2)
O(3B)	2881(4)	-421(3)	6893(2)	43(1)
C(4B)	2634(5)	854(4)	6944(2)	27(2)
O(4B)	2134(3)	748(3)	7345(2)	33(1)
C(5B)	2333(5)	1523(4)	6687(3)	32(2)
O(5B)	2742(3)	1557(3)	6250(2)	36(1)
C(6B)	2545(6)	2225(5)	6918(3)	42(2)
O(6B)	3462(4)	2285(3)	7008(2)	49(2)
C(1C)	-447(5)	1043(5)	5066(3)	34(2)
C(2C)	194(6)	439(5)	4944(3)	37(2)
O(2C)	-283(4)	-203(3)	4847(2)	51(2)
C(3C)	807(6)	320(5)	5350(3)	38(2)
O(3C)	1434(5)	-213(4)	5234(2)	55(2)
C(4C)	1255(5)	1015(4)	5485(3)	34(2)
O(4C)	1640(4)	859(3)	5921(2)	35(1)
C(5C)	582(6)	1611(5)	5551(3)	40(2)
O(5C)	6(4)	1673(3)	5163(2)	37(1)
C(6C)	1009(7)	2351(5)	5582(4)	51(2)
O(6C)	391(6)	2870(4)	5689(3)	73(2)
C(1D)	-3654(6)	787(6)	5757(3)	54(3)
C(2D)	-3355(6)	289(6)	5382(3)	51(2)
O(2D)	-3784(5)	-362(5)	5402(3)	74(2)
C(3D)	-2360(6)	205(5)	5394(3)	43(2)
O(3D)	-2099(4)	-231(4)	5016(2)	57(2)
O(4D)	-987(3)	813(3)	5426(2)	35(1)
C(4D)	-1924(5)	937(5)	5385(3)	42(2)
C(5D)	-2255(6)	1394(6)	5776(3)	49(2)
O(5D)	-3187(4)	1457(4)	5756(2)	55(2)
C(6D)	-1916(7)	2139(5)	5787(4)	56(2)
O(6OD)	-2218(5)	2540(4)	5404(3)	70(2)
C(1E)	-5011(5)	-98(5)	7297(3)	37(2)
C(2E)	-4903(6)	-669(5)	6935(3)	46(2)
O(2OE)	-4779(5)	-1358(3)	7148(3)	59(2)
C(3E)	-4134(6)	-492(5)	6624(3)	45(2)
O(3E)	-4068(6)	-1016(4)	6273(3)	78(2)
C(4E)	-4254(5)	267(5)	6434(3)	41(2)
O(4E)	-3499(4)	430(4)	6172(2)	47(2)
C(5E)	-4360(6)	797(5)	6831(3)	40(2)
O(5E)	-5103(4)	591(3)	7098(2)	38(1)

C(6E)	-4526(6)	1563(6)	6677(3)	52(2)
O(6E)	-5293(5)	1604(4)	6390(2)	58(2)
C(1F)	-3387(5)	-1(4)	8884(3)	33(2)
C(2F)	-3813(6)	-712(5)	8756(3)	36(2)
O(2F)	-3283(4)	-1276(3)	8933(2)	49(2)
C(3F)	-3904(5)	-750(4)	8256(3)	33(2)
O(3F)	-4392(4)	-1390(3)	8123(2)	48(2)
C(4F)	-4380(5)	-98(4)	8063(2)	33(2)
O(4F)	-4256(4)	-123(3)	7584(2)	38(1)
C(5F)	-3995(6)	604(5)	8235(3)	40(2)
O(5F)	-3908(4)	580(3)	8719(2)	39(1)
C(6F)	-4533(6)	1261(5)	8127(3)	45(2)
O(6F)	-5394(4)	1196(4)	8309(3)	57(2)
C(1G)	18(5)	639(4)	8983(3)	31(2)
C(2G)	-324(5)	-57(5)	9193(3)	38(2)
O(2G)	329(4)	-601(4)	9177(2)	51(2)
C(3G)	-1139(5)	-302(4)	8944(3)	32(2)
O(3G)	-1485(4)	-934(3)	9159(2)	48(2)
C(4G)	-1814(5)	282(4)	8943(3)	32(2)
O(4G)	-2544(3)	26(3)	8676(2)	32(1)
C(5G)	-1416(5)	961(4)	8731(3)	37(2)
O(5G)	-654(4)	1172(3)	8983(2)	40(1)
C(6G)	-2041(6)	1616(5)	8746(4)	56(3)
O(6G)	-1568(5)	2222(4)	8572(3)	77(2)
O(1W)	-5477(5)	2709(4)	5781(2)	62(2)
O(2W)	-5792(6)	1843(6)	5006(3)	87(3)
O(3W)	2936(6)	-1857(4)	6622(3)	85(3)
O(4W)	-3758(6)	-2576(5)	7046(3)	90(3)
O(5W)	-3672(6)	3254(5)	5781(3)	82(2)
O(6W)	3719(7)	-1423(6)	8286(4)	100(3)
O(7W)	740(9)	-971(9)	4239(4)	169(6)
O(8W)	3696(9)	-2185(7)	7449(4)	125(4)
O(9W)	2570(8)	2973(8)	8675(6)	154(6)
O(10W)	-5756(6)	153(6)	5232(3)	94(3)
O(11W)	-5673(8)	-689(8)	5331(5)	92(4)
O(12W)	2963(12)	-341(10)	9271(6)	116(6)
O(13W)	2519(15)	-3305(7)	4933(7)	123(7)
O(14W)	-1042(14)	-1784(10)	4418(6)	105(7)
O(15A)	4120(20)	-1601(19)	5906(12)	80(9)
O(15W)	4420(20)	-2015(17)	6032(9)	156(12)
O(16W)	-6508(15)	525(13)	4857(8)	92(7)
O(17W)	-5770(20)	1385(18)	5028(11)	38(8)
O(18W)	2730(30)	1920(50)	9396(14)	210(40)
O(18A)	3170(30)	2190(30)	9357(18)	118(15)
O(20A)	2340(30)	-380(20)	4436(15)	128(13)
O(20C)	2920(30)	-940(30)	4654(18)	93(13)
O(20D)	3330(40)	-1250(40)	4950(20)	130(20)
O(21W)	2990(30)	-1690(30)	8258(16)	82(12)
N(1)	371(9)	-2195(6)	5687(4)	88(3)
C(1)	-112(10)	-1551(7)	5833(4)	81(4)
C(2)	337(12)	-1269(9)	6264(5)	111(5)
C(3)	-78(9)	-639(7)	6426(6)	94(4)
C(4)	-285(8)	-512(7)	6901(4)	72(3)
C(5)	-693(8)	205(7)	6987(4)	66(3)
C(6)	-297(9)	821(8)	6805(5)	86(4)
C(7)	-678(13)	1511(9)	6878(7)	118(6)
C(8)	-1427(11)	1537(9)	7113(6)	100(5)
C(9)	-1832(9)	946(7)	7285(5)	77(3)
C(10)	-1449(7)	257(7)	7228(4)	63(3)
C(11)	-1920(8)	-360(7)	7438(4)	71(3)
C(12)	-1545(8)	-977(8)	7606(4)	78(4)
C(13)	-621(8)	-1127(6)	7611(5)	72(3)

C(14)	-354(10)	-1519(7)	8006(6)	87(4)
C(15)	526(16)	-1686(7)	8037(8)	133(9)
C(16)	1164(9)	-1488(7)	7715(7)	86(4)
C(17)	868(10)	-1119(8)	7317(7)	101(5)
C(18)	14(6)	-959(5)	7272(5)	59(3)
C(19)	-151(14)	-2624(8)	5364(5)	122(7)
C(20)	1220(13)	-2035(9)	5493(6)	119(6)

Table S3: Bond lengths [A] and angles [deg] for cdbzp-16.

C(1A)-O(4B)	1.342(9)
C(1A)-O(5A)	1.360(10)
C(1A)-C(2A)	1.408(11)
C(2A)-O(2A)	1.375(10)
C(2A)-C(3A)	1.523(11)
C(3A)-O(3A)	1.367(9)
C(3A)-C(4A)	1.434(11)
C(4A)-C(5A)	1.429(11)
C(4A)-O(4A)	1.430(10)
O(4A)-C(1G)	1.297(9)
C(5A)-O(5A)	1.441(11)
C(5A)-C(6A)	1.459(12)
C(6A)-O(6A)	1.347(13)
C(1B)-O(5B)	1.343(9)
C(1B)-O(4C)	1.423(10)
C(1B)-C(2B)	1.431(12)
C(2B)-O(2B)	1.359(10)
C(2B)-C(3B)	1.410(11)
C(3B)-O(3B)	1.368(9)
C(3B)-C(4B)	1.420(10)
C(4B)-O(4B)	1.351(8)
C(4B)-C(5B)	1.447(10)
C(5B)-O(5B)	1.351(9)
C(5B)-C(6B)	1.425(11)
C(6B)-O(6B)	1.433(11)
C(1C)-O(5C)	1.335(9)
C(1C)-O(4D)	1.349(9)
C(1C)-C(2C)	1.488(11)
C(2C)-O(2C)	1.372(10)
C(2C)-C(3C)	1.470(11)
C(3C)-O(3C)	1.381(10)
C(3C)-C(4C)	1.451(11)
C(4C)-O(4C)	1.359(9)
C(4C)-C(5C)	1.485(12)
C(5C)-O(5C)	1.385(10)
C(5C)-C(6C)	1.460(12)
C(6C)-O(6C)	1.350(12)
C(1D)-O(4E)	1.319(10)
C(1D)-O(5D)	1.380(12)
C(1D)-C(2D)	1.424(14)
C(2D)-O(2D)	1.325(11)
C(2D)-C(3D)	1.536(13)
C(3D)-O(3D)	1.349(10)
C(3D)-C(4D)	1.453(13)
O(4D)-C(4D)	1.461(10)
C(4D)-C(5D)	1.432(12)
C(5D)-C(6D)	1.410(13)
C(5D)-O(5D)	1.437(11)
C(6D)-O(6OD)	1.345(12)
C(1E)-O(5E)	1.336(10)
C(1E)-O(4F)	1.401(9)
C(1E)-C(2E)	1.420(12)
C(2E)-O(2OE)	1.358(11)
C(2E)-C(3E)	1.488(13)
C(3E)-O(3E)	1.334(10)
C(3E)-C(4E)	1.446(12)
C(4E)-O(4E)	1.392(10)
C(4E)-C(5E)	1.440(12)
C(5E)-O(5E)	1.402(10)

C(5E)-C(6E)	1.437(13)
C(6E)-O(6E)	1.416(11)
C(1F)-O(5F)	1.375(9)
C(1F)-O(4G)	1.415(9)
C(1F)-C(2F)	1.454(11)
C(2F)-O(2F)	1.373(9)
C(2F)-C(3F)	1.376(10)
C(3F)-O(3F)	1.402(10)
C(3F)-C(4F)	1.460(11)
C(4F)-O(4F)	1.323(8)
C(4F)-C(5F)	1.449(12)
C(5F)-O(5F)	1.330(9)
C(5F)-C(6F)	1.451(12)
C(6F)-O(6F)	1.417(11)
C(1G)-O(5G)	1.394(9)
C(1G)-C(2G)	1.452(11)
C(2G)-O(2G)	1.386(10)
C(2G)-C(3G)	1.491(11)
C(3G)-O(3G)	1.366(9)
C(3G)-C(4G)	1.460(11)
C(4G)-O(4G)	1.411(9)
C(4G)-C(5G)	1.463(11)
C(5G)-O(5G)	1.407(10)
C(5G)-C(6G)	1.502(12)
C(6G)-O(6G)	1.376(11)
O(2W)-O(17W)	0.81(3)
O(6W)-O(21W)	1.22(5)
O(10W)-O(11W)	1.512(17)
O(10W)-O(16W)	1.68(2)
O(12W)-O(20A)#1	1.42(4)
O(15A)-O(15W)	0.93(4)
O(18W)-O(18A)	0.84(8)
O(20A)-O(12W)#2	1.42(4)
O(20A)-O(20C)	1.45(6)
O(20C)-O(20D)	1.18(7)
N(1)-C(19)	1.411(18)
N(1)-C(1)	1.413(17)
N(1)-C(20)	1.44(2)
C(1)-C(2)	1.451(18)
C(2)-C(3)	1.354(19)
C(3)-C(4)	1.353(18)
C(4)-C(18)	1.364(16)
C(4)-C(5)	1.429(17)
C(5)-C(10)	1.339(15)
C(5)-C(6)	1.339(16)
C(6)-C(7)	1.36(2)
C(7)-C(8)	1.32(2)
C(8)-C(9)	1.301(17)
C(9)-C(10)	1.357(16)
C(10)-C(11)	1.427(15)
C(11)-C(12)	1.312(16)
C(12)-C(13)	1.444(17)
C(13)-C(14)	1.346(18)
C(13)-C(18)	1.377(17)
C(14)-C(15)	1.39(2)
C(15)-C(16)	1.36(3)
C(16)-C(17)	1.34(2)
C(17)-C(18)	1.347(17)
O(4B)-C(1A)-O(5A)	105.5(6)
O(4B)-C(1A)-C(2A)	108.2(7)
O(5A)-C(1A)-C(2A)	112.5(7)

O(2A)-C(2A)-C(1A)	109.5(7)
O(2A)-C(2A)-C(3A)	113.5(7)
C(1A)-C(2A)-C(3A)	109.5(7)
O(3A)-C(3A)-C(4A)	112.4(6)
O(3A)-C(3A)-C(2A)	105.6(6)
C(4A)-C(3A)-C(2A)	113.0(7)
C(5A)-C(4A)-O(4A)	109.7(7)
C(5A)-C(4A)-C(3A)	109.6(7)
O(4A)-C(4A)-C(3A)	110.2(7)
C(1G)-O(4A)-C(4A)	119.0(6)
C(4A)-C(5A)-O(5A)	106.4(7)
C(4A)-C(5A)-C(6A)	114.7(7)
O(5A)-C(5A)-C(6A)	110.7(8)
C(1A)-O(5A)-C(5A)	115.6(6)
O(6A)-C(6A)-C(5A)	110.2(9)
O(5B)-C(1B)-O(4C)	110.8(6)
O(5B)-C(1B)-C(2B)	110.2(6)
O(4C)-C(1B)-C(2B)	110.2(7)
O(2B)-C(2B)-C(3B)	106.9(7)
O(2B)-C(2B)-C(1B)	109.9(7)
C(3B)-C(2B)-C(1B)	107.6(7)
O(3B)-C(3B)-C(2B)	107.7(7)
O(3B)-C(3B)-C(4B)	109.7(6)
C(2B)-C(3B)-C(4B)	108.8(7)
O(4B)-C(4B)-C(3B)	104.0(6)
O(4B)-C(4B)-C(5B)	108.9(6)
C(3B)-C(4B)-C(5B)	111.7(6)
C(1A)-O(4B)-C(4B)	112.9(6)
O(5B)-C(5B)-C(6B)	104.3(6)
O(5B)-C(5B)-C(4B)	108.4(6)
C(6B)-C(5B)-C(4B)	114.8(6)
C(1B)-O(5B)-C(5B)	110.5(6)
C(5B)-C(6B)-O(6B)	111.3(7)
O(5C)-C(1C)-O(4D)	115.3(6)
O(5C)-C(1C)-C(2C)	107.1(6)
O(4D)-C(1C)-C(2C)	110.7(7)
O(2C)-C(2C)-C(3C)	111.8(7)
O(2C)-C(2C)-C(1C)	106.2(7)
C(3C)-C(2C)-C(1C)	110.9(6)
O(3C)-C(3C)-C(4C)	107.5(7)
O(3C)-C(3C)-C(2C)	111.7(6)
C(4C)-C(3C)-C(2C)	112.0(7)
O(4C)-C(4C)-C(3C)	104.9(6)
O(4C)-C(4C)-C(5C)	109.8(6)
C(3C)-C(4C)-C(5C)	107.3(7)
C(4C)-O(4C)-C(1B)	120.6(6)
O(5C)-C(5C)-C(6C)	105.2(7)
O(5C)-C(5C)-C(4C)	114.0(6)
C(6C)-C(5C)-C(4C)	109.0(7)
C(1C)-O(5C)-C(5C)	114.8(6)
O(6C)-C(6C)-C(5C)	107.5(8)
O(4E)-C(1D)-O(5D)	108.4(7)
O(4E)-C(1D)-C(2D)	105.5(9)
O(5D)-C(1D)-C(2D)	110.9(8)
O(2D)-C(2D)-C(1D)	110.0(8)
O(2D)-C(2D)-C(3D)	114.2(9)
C(1D)-C(2D)-C(3D)	111.4(8)
O(3D)-C(3D)-C(4D)	110.8(7)
O(3D)-C(3D)-C(2D)	109.5(7)
C(4D)-C(3D)-C(2D)	111.8(8)
C(1C)-O(4D)-C(4D)	120.3(6)
C(5D)-C(4D)-C(3D)	108.8(7)

C(5D)-C(4D)-O(4D)	112.1(7)
C(3D)-C(4D)-O(4D)	108.6(7)
C(6D)-C(5D)-C(4D)	114.0(9)
C(6D)-C(5D)-O(5D)	107.3(8)
C(4D)-C(5D)-O(5D)	111.5(8)
C(1D)-O(5D)-C(5D)	116.9(8)
O(6OD)-C(6D)-C(5D)	110.2(9)
O(5E)-C(1E)-O(4F)	110.1(7)
O(5E)-C(1E)-C(2E)	111.9(6)
O(4F)-C(1E)-C(2E)	105.7(7)
O(2OE)-C(2E)-C(1E)	110.6(7)
O(2OE)-C(2E)-C(3E)	108.7(8)
C(1E)-C(2E)-C(3E)	110.0(8)
O(3E)-C(3E)-C(4E)	113.0(7)
O(3E)-C(3E)-C(2E)	109.0(8)
C(4E)-C(3E)-C(2E)	107.3(7)
O(4E)-C(4E)-C(5E)	110.4(7)
O(4E)-C(4E)-C(3E)	105.6(7)
C(5E)-C(4E)-C(3E)	110.1(7)
C(1D)-O(4E)-C(4E)	113.0(7)
O(5E)-C(5E)-C(6E)	104.5(7)
O(5E)-C(5E)-C(4E)	108.5(7)
C(6E)-C(5E)-C(4E)	114.1(7)
C(1E)-O(5E)-C(5E)	111.1(6)
O(6E)-C(6E)-C(5E)	110.9(8)
O(5F)-C(1F)-O(4G)	112.1(6)
O(5F)-C(1F)-C(2F)	107.4(6)
O(4G)-C(1F)-C(2F)	110.2(6)
O(2F)-C(2F)-C(3F)	112.0(7)
O(2F)-C(2F)-C(1F)	105.8(6)
C(3F)-C(2F)-C(1F)	109.0(7)
C(2F)-C(3F)-O(3F)	110.5(7)
C(2F)-C(3F)-C(4F)	111.7(7)
O(3F)-C(3F)-C(4F)	105.7(6)
O(4F)-C(4F)-C(5F)	106.9(7)
O(4F)-C(4F)-C(3F)	104.9(6)
C(5F)-C(4F)-C(3F)	110.4(7)
C(4F)-O(4F)-C(1E)	115.7(6)
O(5F)-C(5F)-C(4F)	109.6(7)
O(5F)-C(5F)-C(6F)	106.5(7)
C(4F)-C(5F)-C(6F)	112.4(7)
C(5F)-O(5F)-C(1F)	114.0(6)
O(6F)-C(6F)-C(5F)	113.4(7)
O(4A)-C(1G)-O(5G)	112.2(6)
O(4A)-C(1G)-C(2G)	109.5(7)
O(5G)-C(1G)-C(2G)	107.5(6)
O(2G)-C(2G)-C(1G)	108.0(7)
O(2G)-C(2G)-C(3G)	113.1(7)
C(1G)-C(2G)-C(3G)	111.6(6)
O(3G)-C(3G)-C(4G)	107.3(6)
O(3G)-C(3G)-C(2G)	111.4(6)
C(4G)-C(3G)-C(2G)	113.1(7)
O(4G)-C(4G)-C(3G)	109.9(6)
O(4G)-C(4G)-C(5G)	112.9(6)
C(3G)-C(4G)-C(5G)	106.2(6)
C(4G)-O(4G)-C(1F)	122.0(5)
O(5G)-C(5G)-C(4G)	111.8(6)
O(5G)-C(5G)-C(6G)	108.4(7)
C(4G)-C(5G)-C(6G)	110.5(7)
C(1G)-O(5G)-C(5G)	116.0(6)
O(6G)-C(6G)-C(5G)	104.4(7)
O(11W)-O(10W)-O(16W)	123.4(12)

O(12W)#2-O(20A)-O(20C)	159(4)
O(20D)-O(20C)-O(20A)	159(6)
C(19)-N(1)-C(1)	107.9(13)
C(19)-N(1)-C(20)	113.0(13)
C(1)-N(1)-C(20)	115.0(12)
N(1)-C(1)-C(2)	104.7(13)
C(3)-C(2)-C(1)	108.8(16)
C(4)-C(3)-C(2)	124.1(15)
C(3)-C(4)-C(18)	122.5(13)
C(3)-C(4)-C(5)	114.0(12)
C(18)-C(4)-C(5)	122.4(11)
C(10)-C(5)-C(6)	121.4(13)
C(10)-C(5)-C(4)	121.5(11)
C(6)-C(5)-C(4)	117.1(12)
C(5)-C(6)-C(7)	118.2(14)
C(8)-C(7)-C(6)	118.5(16)
C(9)-C(8)-C(7)	124.4(16)
C(8)-C(9)-C(10)	117.8(13)
C(5)-C(10)-C(9)	119.6(11)
C(5)-C(10)-C(11)	125.9(12)
C(9)-C(10)-C(11)	114.5(11)
C(12)-C(11)-C(10)	123.2(11)
C(11)-C(12)-C(13)	126.0(11)
C(14)-C(13)-C(18)	115.7(12)
C(14)-C(13)-C(12)	113.7(13)
C(18)-C(13)-C(12)	130.6(12)
C(13)-C(14)-C(15)	117.0(17)
C(16)-C(15)-C(14)	127.5(16)
C(17)-C(16)-C(15)	113.7(15)
C(16)-C(17)-C(18)	120.3(17)
C(17)-C(18)-C(4)	121.1(14)
C(17)-C(18)-C(13)	125.7(14)
C(4)-C(18)-C(13)	112.7(10)

Symmetry transformations used to generate equivalent atoms:
#1 -x+1/2,-y,z+1/2   #2 -x+1/2,-y,z-1/2

Table S4: Anisotropic displacement parameters (A^2 x 10^3) for cdbzp-16. The anisotropic displacement factor exponent takes the form: -2 pi^2 [h^2 a*^2 U11 + ... + 2 h k a* b* U12]

	U11	U22	U33	U23	U13	U12
C(1A)	32(4)	44(5)	38(4)	-2(4)	-2(4)	-5(4)
C(2A)	37(5)	51(5)	35(4)	2(4)	8(4)	0(4)
O(2A)	46(4)	55(4)	48(3)	15(3)	13(3)	15(3)
C(3A)	37(4)	35(4)	34(4)	6(3)	5(4)	5(4)
O(3A)	53(4)	56(4)	50(4)	18(3)	10(3)	15(3)
C(4A)	41(5)	41(5)	30(4)	3(3)	1(4)	-3(4)
O(4A)	35(3)	39(3)	28(3)	1(2)	4(2)	-3(3)
C(5A)	53(5)	35(4)	37(4)	2(3)	12(4)	4(4)
O(5A)	64(4)	32(3)	33(3)	-1(2)	12(3)	-7(3)
C(6A)	76(7)	42(5)	59(6)	10(5)	28(6)	14(5)
O(6A)	117(7)	54(4)	81(6)	-22(4)	36(5)	-6(5)
C(1B)	31(4)	46(5)	30(4)	-5(4)	4(3)	1(4)
C(2B)	45(5)	48(5)	32(4)	-7(4)	0(4)	7(4)
O(2B)	71(5)	52(4)	38(3)	-14(3)	-3(3)	22(3)
C(3B)	33(4)	23(4)	39(4)	0(3)	5(3)	-1(3)
O(3B)	58(4)	33(3)	38(3)	1(2)	-5(3)	5(3)
C(4B)	23(4)	32(4)	26(3)	1(3)	-1(3)	3(3)
O(4B)	30(3)	38(3)	30(3)	1(2)	2(2)	1(2)
C(5B)	35(4)	33(4)	28(4)	1(3)	3(3)	-2(4)
O(5B)	36(3)	40(3)	32(3)	2(2)	-3(2)	-9(3)
C(6B)	59(6)	32(4)	35(4)	4(3)	-2(4)	1(4)
O(6B)	61(4)	40(3)	46(3)	-4(3)	-14(3)	-12(3)
C(1C)	37(5)	43(5)	22(3)	1(3)	0(3)	-3(4)
C(2C)	44(5)	40(4)	28(4)	-5(3)	-6(4)	-4(4)
O(2C)	52(4)	45(4)	55(4)	-19(3)	-5(3)	-4(3)
C(3C)	49(5)	37(4)	29(4)	-5(3)	-6(4)	1(4)
O(3C)	66(4)	47(4)	53(4)	-20(3)	-14(3)	18(3)
C(4C)	39(4)	36(4)	28(4)	2(3)	0(3)	-5(4)
O(4C)	38(3)	44(3)	23(2)	2(2)	-2(2)	1(3)
C(5C)	41(5)	43(5)	35(4)	-5(4)	2(4)	-4(4)
O(5C)	48(3)	34(3)	28(2)	4(2)	-8(2)	-4(3)
C(6C)	60(6)	28(4)	67(6)	-7(4)	-12(5)	-6(4)
O(6C)	87(6)	38(4)	95(6)	-23(4)	-14(5)	9(4)
C(1D)	40(5)	85(7)	36(5)	10(5)	-3(4)	-9(5)
C(2D)	43(5)	71(6)	37(4)	-3(4)	-2(4)	-21(5)
O(2D)	76(5)	98(6)	47(4)	-4(4)	9(4)	-44(5)
C(3D)	48(5)	58(6)	24(4)	-6(4)	2(4)	-4(5)
O(3D)	57(4)	77(5)	39(3)	-19(3)	7(3)	-15(4)
O(4D)	41(3)	43(3)	21(2)	-1(2)	0(2)	-4(3)
C(4D)	33(5)	61(6)	30(4)	6(4)	1(3)	1(4)
C(5D)	43(5)	62(6)	42(5)	-4(4)	3(4)	8(5)
O(5D)	44(4)	77(5)	43(3)	4(3)	8(3)	10(4)
C(6D)	47(6)	47(5)	74(7)	-7(5)	7(5)	4(5)
O(6OD)	71(5)	59(4)	80(5)	13(4)	3(4)	-2(4)
C(1E)	28(4)	55(5)	27(4)	7(4)	-1(3)	7(4)
C(2E)	47(5)	54(5)	36(4)	5(4)	-5(4)	-6(4)
O(2OE)	81(5)	41(3)	56(4)	8(3)	-3(4)	-5(4)
C(3E)	56(6)	48(5)	31(4)	-7(4)	-2(4)	7(5)
O(3E)	109(7)	67(5)	57(4)	-17(4)	17(5)	10(5)
C(4E)	28(4)	60(6)	36(4)	-1(4)	0(3)	6(4)
O(4E)	36(3)	82(4)	23(3)	10(3)	6(2)	3(3)
C(5E)	42(5)	51(5)	25(4)	2(4)	1(4)	-1(4)
O(5E)	39(3)	52(3)	24(2)	7(2)	0(2)	10(3)
C(6E)	47(6)	65(6)	44(5)	9(5)	-5(4)	-7(5)

O(6E)	62(4)	60(4)	51(4)	27(3)	-3(3)	6(4)
C(1F)	37(4)	36(4)	26(3)	2(3)	-3(3)	3(4)
C(2F)	39(4)	44(5)	25(4)	8(3)	-4(3)	6(4)
O(2F)	55(4)	41(3)	50(3)	22(3)	-17(3)	-6(3)
C(3F)	31(4)	38(4)	30(4)	-1(3)	-1(3)	7(4)
O(3F)	61(4)	37(3)	47(3)	-4(3)	-14(3)	5(3)
C(4F)	35(4)	43(4)	22(3)	2(3)	3(3)	10(4)
O(4F)	37(3)	54(3)	24(2)	9(2)	7(2)	12(3)
C(5F)	48(5)	44(5)	30(4)	6(4)	-3(4)	3(4)
O(5F)	43(3)	45(3)	28(3)	-3(2)	3(2)	4(3)
C(6F)	53(6)	34(4)	48(5)	2(4)	-4(4)	11(4)
O(6F)	58(4)	46(4)	65(4)	4(3)	-13(4)	12(3)
C(1G)	25(4)	36(4)	31(4)	-1(3)	-5(3)	-2(3)
C(2G)	34(4)	48(5)	33(4)	14(4)	-2(4)	-1(4)
O(2G)	43(4)	54(4)	56(4)	18(3)	-10(3)	5(3)
C(3G)	38(4)	34(4)	24(3)	7(3)	3(3)	-1(4)
O(3G)	40(3)	38(3)	65(4)	24(3)	-4(3)	1(3)
C(4G)	33(4)	30(4)	32(4)	-1(3)	-3(3)	1(3)
O(4G)	30(3)	38(3)	28(2)	1(2)	1(2)	2(2)
C(5G)	42(5)	30(4)	39(4)	1(3)	-5(4)	9(4)
O(5G)	39(3)	33(3)	47(3)	-9(2)	6(3)	-8(3)
C(6G)	45(5)	37(5)	87(7)	15(5)	4(5)	0(4)
O(6G)	72(5)	36(4)	122(7)	12(4)	-10(5)	2(4)
O(1W)	99(6)	42(3)	44(3)	-1(3)	-1(4)	12(4)
O(2W)	96(7)	95(7)	71(5)	-7(5)	8(5)	-23(6)
O(3W)	103(6)	43(4)	110(6)	-9(4)	-24(5)	-14(4)
O(4W)	96(6)	74(5)	100(6)	-18(5)	-27(5)	14(5)
O(5W)	89(6)	84(5)	72(5)	-9(4)	-11(5)	14(5)
O(6W)	102(7)	97(7)	100(7)	28(5)	11(6)	18(6)
O(7W)	169(11)	265(16)	74(6)	-58(9)	-32(7)	114(12)
O(8W)	149(11)	125(9)	101(8)	7(7)	-36(8)	26(9)
O(9W)	106(9)	134(11)	223(16)	5(11)	19(10)	-66(9)
O(10W)	79(6)	130(8)	72(5)	-22(6)	9(5)	-6(6)
O(11W)	59(7)	116(11)	101(9)	-13(8)	11(7)	16(8)
O(12W)	132(14)	112(13)	104(11)	-12(10)	-76(11)	25(12)
O(13W)	190(18)	38(7)	142(14)	-13(8)	-16(14)	23(10)
O(14W)	154(18)	83(12)	78(11)	33(9)	-48(12)	-22(12)
O(15W)	230(30)	140(20)	106(18)	9(17)	50(20)	-60(20)
O(16W)	93(15)	101(16)	83(14)	14(13)	35(13)	4(14)
O(18W)	80(30)	490(120)	60(20)	-30(40)	0(20)	60(50)
N(1)	143(11)	65(6)	58(6)	3(5)	8(7)	-5(7)
C(1)	133(12)	57(7)	53(6)	8(5)	5(7)	-7(8)
C(2)	156(15)	96(11)	81(9)	-36(8)	8(10)	-14(11)
C(3)	91(10)	57(7)	132(13)	13(8)	5(9)	7(7)
C(4)	71(8)	82(8)	63(7)	-1(7)	5(6)	-29(7)
C(5)	64(7)	78(8)	58(6)	-12(6)	-6(6)	-9(6)
C(6)	73(8)	104(11)	82(8)	23(8)	8(7)	-38(8)
C(7)	132(15)	58(8)	162(17)	-5(10)	-28(14)	-4(10)
C(8)	98(11)	82(10)	121(12)	30(9)	14(10)	-1(9)
C(9)	79(8)	76(8)	77(8)	13(7)	9(7)	6(7)
C(10)	51(6)	83(8)	53(6)	6(6)	4(5)	-12(6)
C(11)	61(7)	79(8)	74(7)	-2(6)	6(6)	-9(7)
C(12)	66(8)	87(9)	80(8)	14(7)	33(7)	-10(7)
C(13)	77(8)	36(5)	102(9)	-7(6)	-14(8)	6(6)
C(14)	91(10)	61(7)	109(10)	-13(7)	-25(9)	21(7)
C(15)	200(20)	37(7)	160(18)	-8(9)	-102(17)	6(11)
C(16)	67(8)	50(7)	141(14)	-19(8)	-29(9)	2(7)
C(17)	86(10)	82(9)	135(13)	-35(10)	-36(10)	4(8)
C(18)	41(6)	35(5)	100(8)	-30(5)	-1(6)	1(5)
C(19)	230(20)	75(9)	64(7)	4(7)	-25(11)	5(12)
C(20)	170(18)	92(11)	93(11)	9(9)	17(12)	26(12)

Table S5: Hydrogen coordinates (x 10^4) and isotropic displacement parameters (A^2 x 10^3) for cdbzp-16.

	x	y	z	U(eq)
H(1A)	3100	1186	7687	46
H(2A)	2916	520	8367	49
H(2OA)	3158	-545	8056	74
H(3A)	1373	-110	7972	42
H(3OA)	1359	-461	8762	79
H(4A)	1446	972	8710	45
H(5A)	866	1088	7752	50
H(6A1)	626	2357	7914	71
H(6A2)	-15	1833	8209	71
H(6OA)	1392	2398	8570	126
H(1B)	2820	1000	5656	43
H(2B)	3543	323	6245	50
H(2OB)	3113	-443	5746	80
H(3B)	1877	92	6619	38
H(3OB)	2863	-357	7190	65
H(4B)	3248	906	7035	33
H(5B)	1703	1492	6637	38
H(6B1)	2234	2263	7226	51
H(6B2)	2360	2642	6711	51
H(6OB)	3546	2549	7250	74
H(1C)	-799	1139	4774	41
H(2C)	518	586	4650	45
H(2OC)	28	-518	4713	76
H(3C)	481	138	5635	46
H(3OC)	1775	-303	5459	83
H(4C)	1684	1162	5237	41
H(5C)	257	1515	5854	47
H(6C1)	1451	2344	5836	62
H(6C2)	1286	2474	5273	62
H(6OC)	153	3201	5878	110
H(1D)	-4277	888	5719	65
H(2D)	-3503	527	5068	61
H(2OD)	-4252	-297	5539	111
H(3D)	-2200	-53	5699	52
H(3OD)	-1570	-194	4982	86
H(4D)	-2048	1189	5072	50
H(5D)	-2102	1146	6086	59
H(6D1)	-2085	2386	6090	67
H(6D2)	-1286	2119	5776	67
H(6D)	-1890	2898	5350	105
H(1E)	-5520	-215	7499	44
H(2E)	-5425	-689	6729	55
H(2E)	-5234	-1498	7277	89
H(3E)	-3608	-505	6827	54
H(3OE)	-4324	-869	6027	117
H(4E)	-4761	283	6217	50
H(5E)	-3847	783	7044	47
H(6E1)	-4589	1886	6963	62
H(6E2)	-4036	1748	6487	62
H(6OE)	-5317	2018	6254	87
H(1F)	-3337	32	9241	40
H(2F)	-4385	-740	8913	43
H(2OF)	-3582	-1611	9057	73
H(3F)	-3328	-776	8102	40
H(3OF)	-4066	-1755	8088	72

H(4F)	-5000	-126	8146	40
H(5F)	-3421	670	8084	48
H(6F1)	-4264	1708	8269	54
H(6F2)	-4556	1331	7776	54
H(6OF)	-5692	1543	8200	85
H(1G)	496	826	9188	37
H(2G)	-461	41	9538	46
H(2OG)	682	-545	9398	77
H(3G)	-998	-427	8603	38
H(3OG)	-1105	-1258	9190	71
H(4G)	-1990	389	9281	38
H(5G)	-1266	856	8389	44
H(6G1)	-2543	1518	8540	68
H(6G2)	-2238	1710	9078	68
H(6OG)	-1900	2572	8503	115
H(1C1)	-707	-1689	5911	97
H(2C1)	-118	-1171	5576	97
H(1C2)	339	-1655	6517	134
H(2C2)	936	-1147	6182	134
H(3)	-231	-272	6198	112
H(6)	224	779	6633	103
H(7)	-415	1953	6765	141
H(8)	-1684	2010	7158	120
H(9)	-2367	993	7442	92
H(11)	-2523	-324	7457	86
H(12)	-1907	-1351	7733	93
H(14)	-742	-1671	8249	104
H(15)	701	-1966	8308	159
H(16)	1750	-1597	7766	103
H(17)	1255	-974	7073	121
H(191)	-704	-2725	5515	183
H(192)	134	-3095	5290	183
H(193)	-240	-2343	5067	183
H(201)	1540	-1732	5724	178
H(202)	1164	-1763	5191	178
H(203)	1524	-2503	5437	178

Table S6: Torsion angles [deg] for cdbzp-16.

O(4B)-C(1A)-C(2A)-O(2A)	57.1(9)
O(5A)-C(1A)-C(2A)-O(2A)	173.3(7)
O(4B)-C(1A)-C(2A)-C(3A)	-68.0(8)
O(5A)-C(1A)-C(2A)-C(3A)	48.2(9)
O(2A)-C(2A)-C(3A)-O(3A)	64.9(8)
C(1A)-C(2A)-C(3A)-O(3A)	-172.4(7)
O(2A)-C(2A)-C(3A)-C(4A)	-171.8(7)
C(1A)-C(2A)-C(3A)-C(4A)	-49.1(9)
O(3A)-C(3A)-C(4A)-C(5A)	175.4(7)
C(2A)-C(3A)-C(4A)-C(5A)	56.0(10)
O(3A)-C(3A)-C(4A)-O(4A)	-63.8(9)
C(2A)-C(3A)-C(4A)-O(4A)	176.8(6)
C(5A)-C(4A)-O(4A)-C(1G)	-114.8(8)
C(3A)-C(4A)-O(4A)-C(1G)	124.4(7)
O(4A)-C(4A)-C(5A)-O(5A)	-179.9(6)
C(3A)-C(4A)-C(5A)-O(5A)	-58.8(9)
O(4A)-C(4A)-C(5A)-C(6A)	57.2(11)
C(3A)-C(4A)-C(5A)-C(6A)	178.4(8)
O(4B)-C(1A)-O(5A)-C(5A)	59.4(8)
C(2A)-C(1A)-O(5A)-C(5A)	-58.4(9)
C(4A)-C(5A)-O(5A)-C(1A)	62.2(8)
C(6A)-C(5A)-O(5A)-C(1A)	-172.5(7)
C(4A)-C(5A)-C(6A)-O(6A)	54.8(12)
O(5A)-C(5A)-C(6A)-O(6A)	-65.7(10)
O(5B)-C(1B)-C(2B)-O(2B)	179.2(7)
O(4C)-C(1B)-C(2B)-O(2B)	56.7(9)
O(5B)-C(1B)-C(2B)-C(3B)	63.2(9)
O(4C)-C(1B)-C(2B)-C(3B)	-59.3(8)
O(2B)-C(2B)-C(3B)-O(3B)	65.9(8)
C(1B)-C(2B)-C(3B)-O(3B)	-176.0(7)
O(2B)-C(2B)-C(3B)-C(4B)	-175.2(7)
C(1B)-C(2B)-C(3B)-C(4B)	-57.2(9)
O(3B)-C(3B)-C(4B)-O(4B)	-69.6(8)
C(2B)-C(3B)-C(4B)-O(4B)	172.8(6)
O(3B)-C(3B)-C(4B)-C(5B)	173.2(7)
C(2B)-C(3B)-C(4B)-C(5B)	55.6(9)
O(5A)-C(1A)-O(4B)-C(4B)	118.5(6)
C(2A)-C(1A)-O(4B)-C(4B)	-120.9(7)
C(3B)-C(4B)-O(4B)-C(1A)	139.6(7)
C(5B)-C(4B)-O(4B)-C(1A)	-101.3(7)
O(4B)-C(4B)-C(5B)-O(5B)	-170.4(6)
C(3B)-C(4B)-C(5B)-O(5B)	-56.2(8)
O(4B)-C(4B)-C(5B)-C(6B)	73.4(8)
C(3B)-C(4B)-C(5B)-C(6B)	-172.3(7)
O(4C)-C(1B)-O(5B)-C(5B)	56.2(8)
C(2B)-C(1B)-O(5B)-C(5B)	-66.0(8)
C(6B)-C(5B)-O(5B)-C(1B)	-176.6(7)
C(4B)-C(5B)-O(5B)-C(1B)	60.6(8)
O(5B)-C(5B)-C(6B)-O(6B)	-63.7(8)
C(4B)-C(5B)-C(6B)-O(6B)	54.7(9)
O(5C)-C(1C)-C(2C)-O(2C)	179.9(6)
O(4D)-C(1C)-C(2C)-O(2C)	53.5(8)
O(5C)-C(1C)-C(2C)-C(3C)	58.2(8)
O(4D)-C(1C)-C(2C)-C(3C)	-68.2(9)
O(2C)-C(2C)-C(3C)-O(3C)	64.7(9)
C(1C)-C(2C)-C(3C)-O(3C)	-177.0(7)
O(2C)-C(2C)-C(3C)-C(4C)	-174.6(7)
C(1C)-C(2C)-C(3C)-C(4C)	-56.3(9)
O(3C)-C(3C)-C(4C)-O(4C)	-69.9(8)

C(2C)-C(3C)-C(4C)-O(4C)	167.0(7)
O(3C)-C(3C)-C(4C)-C(5C)	173.3(6)
C(2C)-C(3C)-C(4C)-C(5C)	50.2(9)
C(3C)-C(4C)-O(4C)-C(1B)	122.8(7)
C(5C)-C(4C)-O(4C)-C(1B)	-122.2(7)
O(5B)-C(1B)-O(4C)-C(4C)	106.6(7)
C(2B)-C(1B)-O(4C)-C(4C)	-131.2(7)
O(4C)-C(4C)-C(5C)-O(5C)	-164.3(6)
C(3C)-C(4C)-C(5C)-O(5C)	-50.8(9)
O(4C)-C(4C)-C(5C)-C(6C)	78.6(9)
C(3C)-C(4C)-C(5C)-C(6C)	-167.9(7)
O(4D)-C(1C)-O(5C)-C(5C)	63.4(9)
C(2C)-C(1C)-O(5C)-C(5C)	-60.3(8)
C(6C)-C(5C)-O(5C)-C(1C)	178.9(7)
C(4C)-C(5C)-O(5C)-C(1C)	59.6(9)
O(5C)-C(5C)-C(6C)-O(6C)	63.2(9)
C(4C)-C(5C)-C(6C)-O(6C)	-174.2(8)
O(4E)-C(1D)-C(2D)-O(2D)	60.1(10)
O(5D)-C(1D)-C(2D)-O(2D)	177.3(8)
O(4E)-C(1D)-C(2D)-C(3D)	-67.6(10)
O(5D)-C(1D)-C(2D)-C(3D)	49.6(11)
O(2D)-C(2D)-C(3D)-O(3D)	58.3(10)
C(1D)-C(2D)-C(3D)-O(3D)	-176.2(8)
O(2D)-C(2D)-C(3D)-C(4D)	-178.4(7)
C(1D)-C(2D)-C(3D)-C(4D)	-53.0(10)
O(5C)-C(1C)-O(4D)-C(4D)	104.0(8)
C(2C)-C(1C)-O(4D)-C(4D)	-134.3(7)
O(3D)-C(3D)-C(4D)-C(5D)	176.9(8)
C(2D)-C(3D)-C(4D)-C(5D)	54.5(9)
O(3D)-C(3D)-C(4D)-O(4D)	-60.8(9)
C(2D)-C(3D)-C(4D)-O(4D)	176.7(6)
C(1C)-O(4D)-C(4D)-C(5D)	-120.3(8)
C(1C)-O(4D)-C(4D)-C(3D)	119.5(7)
C(3D)-C(4D)-C(5D)-C(6D)	-176.5(8)
O(4D)-C(4D)-C(5D)-C(6D)	63.4(11)
C(3D)-C(4D)-C(5D)-O(5D)	-54.7(10)
O(4D)-C(4D)-C(5D)-O(5D)	-174.8(7)
O(4E)-C(1D)-O(5D)-C(5D)	62.4(10)
C(2D)-C(1D)-O(5D)-C(5D)	-53.0(10)
C(6D)-C(5D)-O(5D)-C(1D)	-178.2(8)
C(4D)-C(5D)-O(5D)-C(1D)	56.3(10)
C(4D)-C(5D)-C(6D)-O(6OD)	69.2(11)
O(5D)-C(5D)-C(6D)-O(6OD)	-54.8(11)
O(5E)-C(1E)-C(2E)-O(2OE)	177.8(7)
O(4F)-C(1E)-C(2E)-O(2OE)	57.9(9)
O(5E)-C(1E)-C(2E)-C(3E)	57.7(9)
O(4F)-C(1E)-C(2E)-C(3E)	-62.1(8)
O(2OE)-C(2E)-C(3E)-O(3E)	61.7(9)
C(1E)-C(2E)-C(3E)-O(3E)	-177.1(7)
O(2OE)-C(2E)-C(3E)-C(4E)	-175.6(7)
C(1E)-C(2E)-C(3E)-C(4E)	-54.4(9)
O(3E)-C(3E)-C(4E)-O(4E)	-63.5(10)
C(2E)-C(3E)-C(4E)-O(4E)	176.3(7)
O(3E)-C(3E)-C(4E)-C(5E)	177.3(8)
C(2E)-C(3E)-C(4E)-C(5E)	57.1(9)
O(5D)-C(1D)-O(4E)-C(4E)	123.8(8)
C(2D)-C(1D)-O(4E)-C(4E)	-117.3(8)
C(5E)-C(4E)-O(4E)-C(1D)	-102.2(9)
C(3E)-C(4E)-O(4E)-C(1D)	138.8(8)
O(4E)-C(4E)-C(5E)-O(5E)	-177.2(6)
C(3E)-C(4E)-C(5E)-O(5E)	-61.0(9)
O(4E)-C(4E)-C(5E)-C(6E)	66.7(10)

C(3E)-C(4E)-C(5E)-C(6E)	-177.1(8)
O(4F)-C(1E)-O(5E)-C(5E)	55.4(7)
C(2E)-C(1E)-O(5E)-C(5E)	-61.8(9)
C(6E)-C(5E)-O(5E)-C(1E)	-175.4(7)
C(4E)-C(5E)-O(5E)-C(1E)	62.5(8)
O(5E)-C(5E)-C(6E)-O(6E)	-59.1(9)
C(4E)-C(5E)-C(6E)-O(6E)	59.3(11)
O(5F)-C(1F)-C(2F)-O(2F)	-179.0(6)
O(4G)-C(1F)-C(2F)-O(2F)	58.6(7)
O(5F)-C(1F)-C(2F)-C(3F)	60.4(8)
O(4G)-C(1F)-C(2F)-C(3F)	-62.0(8)
O(2F)-C(2F)-C(3F)-O(3F)	69.4(9)
C(1F)-C(2F)-C(3F)-O(3F)	-173.9(6)
O(2F)-C(2F)-C(3F)-C(4F)	-173.2(7)
C(1F)-C(2F)-C(3F)-C(4F)	-56.4(9)
C(2F)-C(3F)-C(4F)-O(4F)	167.0(7)
O(3F)-C(3F)-C(4F)-O(4F)	-72.8(8)
C(2F)-C(3F)-C(4F)-C(5F)	52.1(9)
O(3F)-C(3F)-C(4F)-C(5F)	172.3(7)
C(5F)-C(4F)-O(4F)-C(1E)	-115.2(8)
C(3F)-C(4F)-O(4F)-C(1E)	127.5(7)
O(5E)-C(1E)-O(4F)-C(4F)	104.1(7)
C(2E)-C(1E)-O(4F)-C(4F)	-134.8(7)
O(4F)-C(4F)-C(5F)-O(5F)	-165.0(7)
C(3F)-C(4F)-C(5F)-O(5F)	-51.4(9)
O(4F)-C(4F)-C(5F)-C(6F)	76.8(9)
C(3F)-C(4F)-C(5F)-C(6F)	-169.6(7)
C(4F)-C(5F)-O(5F)-C(1F)	60.2(9)
C(6F)-C(5F)-O(5F)-C(1F)	-177.9(7)
O(4G)-C(1F)-O(5F)-C(5F)	56.8(9)
C(2F)-C(1F)-O(5F)-C(5F)	-64.3(8)
O(5F)-C(5F)-C(6F)-O(6F)	-65.1(10)
C(4F)-C(5F)-C(6F)-O(6F)	55.0(10)
C(4A)-O(4A)-C(1G)-O(5G)	111.0(7)
C(4A)-O(4A)-C(1G)-C(2G)	-129.7(7)
O(4A)-C(1G)-C(2G)-O(2G)	55.4(8)
O(5G)-C(1G)-C(2G)-O(2G)	177.6(6)
O(4A)-C(1G)-C(2G)-C(3G)	-69.6(8)
O(5G)-C(1G)-C(2G)-C(3G)	52.6(8)
O(2G)-C(2G)-C(3G)-O(3G)	61.3(9)
C(1G)-C(2G)-C(3G)-O(3G)	-176.6(7)
O(2G)-C(2G)-C(3G)-C(4G)	-177.6(6)
C(1G)-C(2G)-C(3G)-C(4G)	-55.5(9)
O(3G)-C(3G)-C(4G)-O(4G)	-60.2(8)
C(2G)-C(3G)-C(4G)-O(4G)	176.5(6)
O(3G)-C(3G)-C(4G)-C(5G)	177.4(6)
C(2G)-C(3G)-C(4G)-C(5G)	54.1(8)
C(3G)-C(4G)-O(4G)-C(1F)	123.9(7)
C(5G)-C(4G)-O(4G)-C(1F)	-117.7(7)
O(5F)-C(1F)-O(4G)-C(4G)	104.4(7)
C(2F)-C(1F)-O(4G)-C(4G)	-136.0(7)
O(4G)-C(4G)-C(5G)-O(5G)	-175.7(6)
C(3G)-C(4G)-C(5G)-O(5G)	-55.2(8)
O(4G)-C(4G)-C(5G)-C(6G)	63.5(9)
C(3G)-C(4G)-C(5G)-C(6G)	-176.0(7)
O(4A)-C(1G)-O(5G)-C(5G)	63.1(8)
C(2G)-C(1G)-O(5G)-C(5G)	-57.3(8)
C(4G)-C(5G)-O(5G)-C(1G)	61.1(8)
C(6G)-C(5G)-O(5G)-C(1G)	-176.9(7)
O(5G)-C(5G)-C(6G)-O(6G)	53.0(10)
C(4G)-C(5G)-C(6G)-O(6G)	175.8(8)
O(12W)#2-O(20A)-O(20C)-O(20D)	65(21)

C(19)-N(1)-C(1)-C(2)	163.4(12)
C(20)-N(1)-C(1)-C(2)	-69.4(14)
N(1)-C(1)-C(2)-C(3)	179.2(12)
C(1)-C(2)-C(3)-C(4)	133.7(15)
C(2)-C(3)-C(4)-C(18)	9(2)
C(2)-C(3)-C(4)-C(5)	177.8(14)
C(3)-C(4)-C(5)-C(10)	127.5(13)
C(18)-C(4)-C(5)-C(10)	-64.1(16)
C(3)-C(4)-C(5)-C(6)	-51.0(16)
C(18)-C(4)-C(5)-C(6)	117.5(13)
C(10)-C(5)-C(6)-C(7)	1(2)
C(4)-C(5)-C(6)-C(7)	179.4(13)
C(5)-C(6)-C(7)-C(8)	-2(2)
C(6)-C(7)-C(8)-C(9)	1(3)
C(7)-C(8)-C(9)-C(10)	1(2)
C(6)-C(5)-C(10)-C(9)	1.0(18)
C(4)-C(5)-C(10)-C(9)	-177.4(12)
C(6)-C(5)-C(10)-C(11)	-179.6(12)
C(4)-C(5)-C(10)-C(11)	2.0(18)
C(8)-C(9)-C(10)-C(5)	-2.2(19)
C(8)-C(9)-C(10)-C(11)	178.4(13)
C(5)-C(10)-C(11)-C(12)	30.0(19)
C(9)-C(10)-C(11)-C(12)	-150.6(13)
C(10)-C(11)-C(12)-C(13)	1(2)
C(11)-C(12)-C(13)-C(14)	144.4(13)
C(11)-C(12)-C(13)-C(18)	-38(2)
C(18)-C(13)-C(14)-C(15)	2.1(17)
C(12)-C(13)-C(14)-C(15)	179.9(12)
C(13)-C(14)-C(15)-C(16)	1(2)
C(14)-C(15)-C(16)-C(17)	-3(2)
C(15)-C(16)-C(17)-C(18)	1.9(19)
C(16)-C(17)-C(18)-C(4)	172.4(12)
C(16)-C(17)-C(18)-C(13)	1.4(19)
C(3)-C(4)-C(18)-C(17)	51.2(17)
C(5)-C(4)-C(18)-C(17)	-116.3(14)
C(3)-C(4)-C(18)-C(13)	-136.8(13)
C(5)-C(4)-C(18)-C(13)	55.8(14)
C(14)-C(13)-C(18)-C(17)	-3.5(17)
C(12)-C(13)-C(18)-C(17)	179.1(13)
C(14)-C(13)-C(18)-C(4)	-175.2(10)
C(12)-C(13)-C(18)-C(4)	7.5(18)

Symmetry transformations used to generate equivalent atoms:
#1 -x+1/2,-y,z+1/2 #2 -x+1/2,-y,z-1/2

Table S7: Hydrogen bonds for cdbzp-16 [A and deg.].

D-H...A	d(D-H)	d(H...A)	d(D...A)	<(DHA)
O(3A)-H(3OA)...O(2G)	0.82	1.96	2.781(9)	176.9
O(3A)-H(3OA)...O(4A)	0.82	2.42	2.811(8)	110.5
O(3B)-H(3OB)...O(2A)	0.82	1.85	2.659(8)	169.7
O(3B)-H(3OB)...O(4B)	0.82	2.28	2.660(8)	108.4
O(3C)-H(3OC)...O(2B)	0.82	1.94	2.752(9)	168.5
O(6C)-H(6OC)...O(2G)#3	0.86	2.24	2.933(10)	137.7
O(3D)-H(3OD)...O(2C)	0.82	2.01	2.827(10)	173.4
O(3D)-H(3OD)...O(4D)	0.82	2.33	2.748(8)	112.6
O(3E)-H(3OE)...O(2D)	0.82	2.10	2.680(11)	127.7
O(3F)-H(3OF)...O(6B)#4	0.82	1.95	2.759(8)	170.8
O(3G)-H(3OG)...O(6C)#4	0.82	1.92	2.725(10)	169.2
C(4B)-H(4B)...O(5E)#5	0.98	2.60	3.532(9)	159.2
C(1E)-H(1E)...O(2A)#6	0.98	2.42	3.369(10)	163.2
C(1F)-H(1F)...O(3D)#7	0.98	2.25	3.207(10)	165.9
C(1)-H(1C1)...O(6A)#4	0.97	2.26	2.823(14)	116.0

Symmetry transformations used to generate equivalent atoms:
#1 -x+1/2,-y,z+1/2 #2 -x+1/2,-y,z-1/2 #3 -x,y+1/2,-z+3/2
#4 -x,y-1/2,-z+3/2 #5 x+1,y,z #6 x-1,y,z
#7 -x-1/2,-y,z+1/2

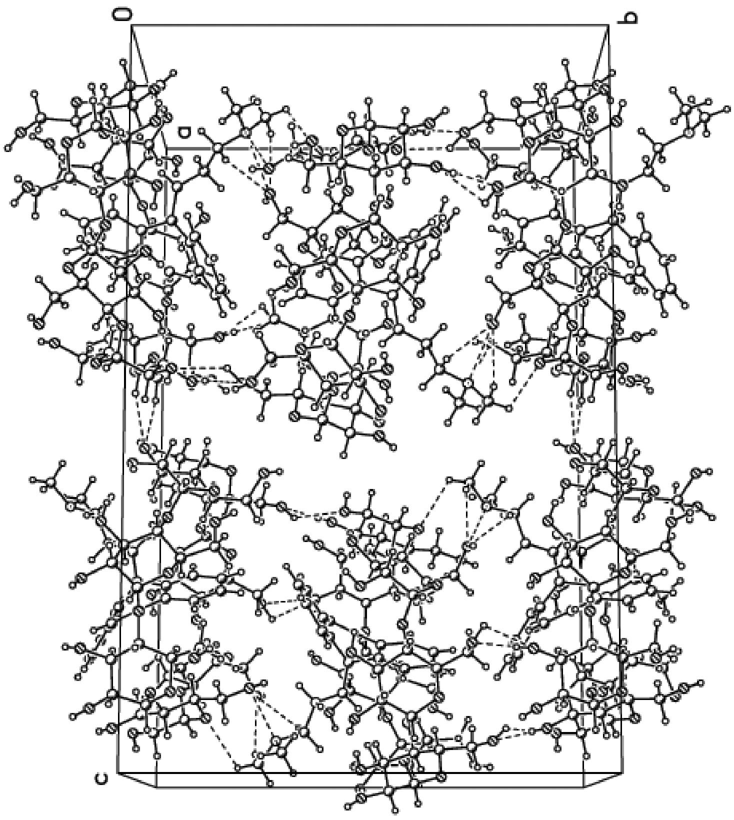

Figure S2: Packing diagram of complex **1**.

Crystallographic data for amitryptiline (**2**)

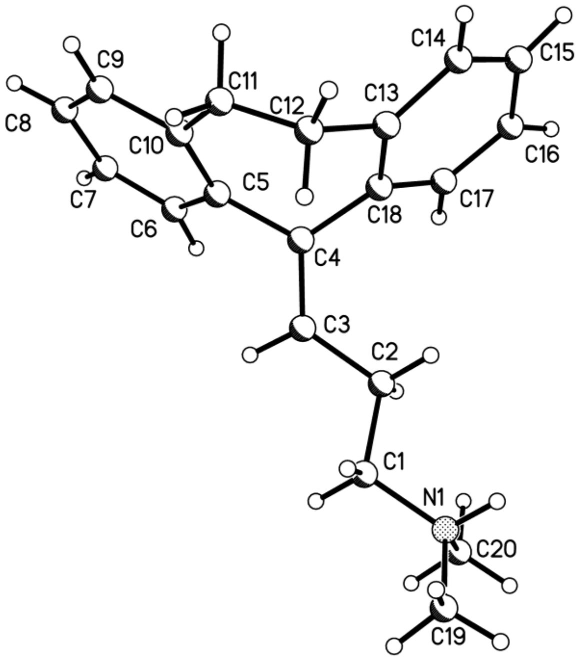

Figure S3: The structure of compound **2** in the inclusion complex with the atom numbering scheme from the X-ray results.

Table S8: Crystal data and structure refinement for cdamtr-16.

Identification code	cdamtr-16
Empirical formula	C62 H93 N O48.10
Formula weight	1621.97
Temperature	293(2) K
Wavelength	0.71073 Å
Crystal system, space group	orthorhombic, P212121
Unit cell dimensions	a = 15.1868(15)Å alpha = 90 deg.
	b = 18.5587(18)Å beta = 90 deg.
	c = 29.501(3) Å gamma = 90 deg.
Volume	8314.7(14) A^3
Z, Calculated density	4, 1.296 Mg/m^3
Absorption coefficient	0.113 mm^-1
F(000)	3427
Crystal size	0.38 x 0.35 x 0.06 mm
Theta range for data collection	1.76 to 30.00 deg.
Limiting indices	-21<=h<=21, -26<=k<=26, -26<=l<=26
Reflections collected / unique	110431 / 20064 [R(int) = 0.0527]
Completeness to theta	30.00 82.6 %
Refinement method	Full-matrix least-squares on F^2
Data / restraints / parameters	20064 / 0 / 1102
Goodness-of-fit on F^2	1.028
Final R indices [I>2sigma(I)]	R1 = 0.0680, wR2 = 0.1830
R indices (all data)	R1 = 0.0895, wR2 = 0.1972
Absolute structure parameter	0.4(7)
Largest diff. peak and hole	0.551 and -0.348 e.A^-3

Table S9: Atomic coordinates (x 10^4) and equivalent isotropic displacement parameters (A^2 x 10^3) for cdamtr-16. U(eq) is defined as one third of the trace of the orthogonalized Uij tensor.

	x	y	z	U(eq)
C(1A)	2507(2)	990(2)	7765(1)	39(1)
C(2A)	2577(2)	355(2)	8092(1)	40(1)
O(2A)	3024(1)	-218(1)	7864(1)	52(1)
C(3A)	1679(2)	122(1)	8262(1)	38(1)
O(3A)	1802(1)	-403(1)	8606(1)	54(1)
C(4A)	1169(2)	769(1)	8436(1)	34(1)
O(4A)	311(1)	513(1)	8558(1)	35(1)
C(5A)	1114(2)	1332(1)	8068(1)	40(1)
O(5A)	2002(1)	1546(1)	7954(1)	46(1)
C(6A)	603(3)	1999(2)	8197(1)	59(1)
O(6A)	877(2)	2296(2)	8611(1)	82(1)
C(1B)	2554(2)	950(2)	5983(1)	40(1)
C(2B)	2900(2)	267(2)	6212(1)	43(1)
O(2B)	2705(2)	-350(1)	5939(1)	57(1)
C(3B)	2485(2)	182(1)	6673(1)	36(1)
O(3B)	2860(1)	-420(1)	6901(1)	46(1)
C(4B)	2623(2)	858(1)	6949(1)	32(1)
O(4B)	2125(1)	757(1)	7357(1)	37(1)
C(5B)	2323(2)	1531(1)	6696(1)	35(1)
O(5B)	2736(1)	1556(1)	6258(1)	41(1)
C(6B)	2556(2)	2232(1)	6927(1)	44(1)
O(6B)	3465(2)	2289(1)	7017(1)	52(1)
C(1C)	-457(2)	1037(1)	5065(1)	36(1)
C(2C)	191(2)	447(2)	4951(1)	41(1)
O(2C)	-295(2)	-193(1)	4849(1)	54(1)
C(3C)	807(2)	322(1)	5345(1)	38(1)
O(3C)	1449(2)	-205(1)	5227(1)	57(1)
C(4C)	1251(2)	1018(1)	5495(1)	36(1)
O(4C)	1638(1)	868(1)	5923(1)	39(1)
C(5C)	580(2)	1624(1)	5553(1)	37(1)
O(5C)	9(1)	1678(1)	5170(1)	41(1)
C(6C)	1031(2)	2356(2)	5598(1)	53(1)
O(6C)	386(2)	2876(1)	5704(1)	69(1)
C(1D)	-3645(2)	812(2)	5768(1)	52(1)
C(2D)	-3361(2)	321(2)	5372(1)	56(1)
O(2D)	-3828(2)	-325(2)	5380(1)	82(1)
C(3D)	-2369(2)	214(2)	5393(1)	44(1)
O(3D)	-2101(2)	-211(2)	5017(1)	58(1)
O(4D)	-987(1)	817(1)	5430(1)	39(1)
C(4D)	-1924(2)	943(2)	5392(1)	40(1)
C(5D)	-2243(2)	1387(2)	5791(1)	46(1)
O(5D)	-3182(1)	1459(1)	5769(1)	56(1)
C(6D)	-1900(2)	2148(2)	5801(2)	62(1)
O(6OD)	-2206(2)	2542(2)	5419(1)	79(1)
C(1E)	-5027(2)	-99(2)	7297(1)	39(1)
C(2E)	-4934(2)	-678(2)	6935(1)	44(1)
O(2OE)	-4813(2)	-1360(1)	7141(1)	64(1)
C(3E)	-4168(2)	-501(2)	6617(1)	45(1)
O(3E)	-4147(2)	-1003(2)	6251(1)	73(1)
C(4E)	-4280(2)	263(2)	6438(1)	40(1)
O(4E)	-3520(1)	422(1)	6174(1)	50(1)
C(5E)	-4376(2)	798(2)	6828(1)	40(1)
O(5E)	-5112(1)	586(1)	7101(1)	41(1)
C(6E)	-4532(2)	1563(2)	6683(1)	49(1)

O(6E)	-5289(2)	1624(1)	6396(1)	58(1)
C(1F)	-3378(2)	3(1)	8878(1)	35(1)
C(2F)	-3803(2)	-714(1)	8755(1)	38(1)
O(2F)	-3276(1)	-1281(1)	8928(1)	51(1)
C(3F)	-3909(2)	-765(1)	8251(1)	38(1)
O(3F)	-4394(1)	-1398(1)	8125(1)	51(1)
C(4F)	-4387(2)	-109(1)	8059(1)	34(1)
O(4F)	-4274(1)	-137(1)	7579(1)	40(1)
C(5F)	-3990(2)	592(1)	8237(1)	39(1)
O(5F)	-3903(1)	578(1)	8717(1)	39(1)
C(6F)	-4522(2)	1257(2)	8118(1)	48(1)
O(6F)	-5383(2)	1201(1)	8303(1)	58(1)
C(1G)	14(2)	658(1)	8999(1)	36(1)
C(2G)	-322(2)	-47(2)	9207(1)	39(1)
O(2G)	343(1)	-583(1)	9198(1)	53(1)
C(3G)	-1128(2)	-307(1)	8956(1)	35(1)
O(3G)	-1481(1)	-929(1)	9175(1)	49(1)
C(4G)	-1814(2)	286(1)	8948(1)	33(1)
O(4G)	-2537(1)	22(1)	8677(1)	36(1)
C(5G)	-1421(2)	968(1)	8736(1)	37(1)
O(5G)	-661(1)	1174(1)	9001(1)	41(1)
C(6G)	-2044(2)	1601(2)	8757(2)	62(1)
O(6G)	-1581(2)	2217(1)	8584(1)	75(1)
O(1W)	3777(2)	2430(2)	7958(1)	86(1)
O(2W)	4523(2)	2719(1)	5782(1)	64(1)
O(3W)	6329(2)	3268(2)	5774(1)	83(1)
O(4W)	2916(2)	-1851(2)	6622(2)	97(1)
O(5W)	3735(3)	-1422(2)	8295(1)	71(1)
O(6W)	3709(4)	-2151(3)	7451(2)	81(1)
O(7W)	4006(6)	6798(5)	5603(3)	88(2)
O(8W)	4061(8)	-1514(10)	5890(6)	111(6)
O(9W)	4244(7)	1507(7)	5026(4)	62(3)
O(9')	4194(4)	1871(2)	5018(2)	63(1)
O(10W)	7442(2)	8082(2)	6203(3)	73(2)
O(10')	7318(9)	7905(8)	6446(7)	62(4)
O(11W)	2050(5)	355(4)	4282(3)	133(2)
O(12W)	7524(16)	8321(6)	5017(4)	97(4)
O(12A)	7730(20)	8252(16)	5144(13)	83(8)
O(12B)	7290(20)	8299(15)	5265(12)	116(9)
O(13W)	5801(4)	5891(4)	5777(2)	81(2)
O(13')	5382(9)	6289(7)	5719(5)	59(3)
O(14W)	4244(2)	165(3)	5239(2)	77(1)
O(14B)	3405(11)	504(9)	4847(6)	68(4)
O(14C)	4306(9)	-743(8)	5355(5)	50(3)
O(15W)	4253(14)	8166(11)	5948(7)	102(6)
O(15')	4508(13)	7900(12)	6070(7)	89(5)
O(16W)	7053(10)	7057(9)	5608(5)	75(4)
O(16')	7273(13)	6559(12)	5662(7)	100(5)
O(17W)	4349(13)	-487(13)	5327(7)	79(5)
O(18W)	2326(15)	-360(12)	4393(8)	108(6)
O(19W)	3691(14)	440(11)	4988(7)	86(5)
O(20W)	3045(16)	-1598(13)	8303(8)	55(5)
O(21W)	3370(30)	-1270(20)	5032(13)	94(10)
O(14')	3020(20)	-978(18)	4715(12)	82(8)
N(1)	441(3)	-2199(2)	5670(1)	82(1)
C(1)	-34(3)	-1546(2)	5830(2)	75(1)
C(2)	394(3)	-1267(2)	6255(2)	75(1)
C(3)	-85(3)	-616(2)	6434(2)	74(1)
C(4)	-288(2)	-472(2)	6867(1)	59(1)
C(5)	-697(2)	229(2)	6975(1)	60(1)
C(6)	-288(3)	845(2)	6808(2)	91(2)
C(7)	-566(5)	1517(3)	6908(3)	135(3)

C(8)	-1318(6)	1605(3)	7158(3)	142(3)
C(9)	-1725(4)	1019(3)	7332(2)	104(2)
C(10)	-1448(3)	319(3)	7240(2)	72(1)
C(11)	-1935(3)	-262(4)	7469(3)	109(2)
C(12)	-1681(3)	-997(3)	7445(3)	119(3)
C(13)	-697(3)	-1158(2)	7565(2)	72(1)
C(14)	-463(4)	-1495(2)	7963(2)	90(2)
C(15)	425(6)	-1633(3)	8050(3)	122(3)
C(16)	1065(4)	-1446(3)	7720(2)	95(2)
C(17)	819(3)	-1104(2)	7356(2)	76(1)
C(18)	-49(2)	-934(2)	7252(2)	63(1)
C(19)	-81(6)	-2588(3)	5346(2)	128(3)
C(20)	1336(5)	-2019(3)	5476(2)	119(2)

Table S10: Bond lengths [A] and angles [deg] for cdamtr-16.

C(1A)-O(5A)	1.401(3)
C(1A)-O(4B)	1.405(3)
C(1A)-C(2A)	1.527(4)
C(2A)-O(2A)	1.431(4)
C(2A)-C(3A)	1.516(4)
C(3A)-O(3A)	1.419(3)
C(3A)-C(4A)	1.517(4)
C(4A)-O(4A)	1.433(3)
C(4A)-C(5A)	1.508(4)
O(4A)-C(1G)	1.403(3)
C(5A)-O(5A)	1.445(3)
C(5A)-C(6A)	1.509(4)
C(6A)-O(6A)	1.405(5)
C(1B)-O(4C)	1.411(3)
C(1B)-O(5B)	1.416(4)
C(1B)-C(2B)	1.530(4)
C(2B)-O(2B)	1.431(4)
C(2B)-C(3B)	1.506(4)
C(3B)-O(3B)	1.422(3)
C(3B)-C(4B)	1.510(4)
C(4B)-O(4B)	1.433(3)
C(4B)-C(5B)	1.525(4)
C(5B)-O(5B)	1.436(4)
C(5B)-C(6B)	1.510(4)
C(6B)-O(6B)	1.411(4)
C(1C)-O(4D)	1.404(3)
C(1C)-O(5C)	1.418(3)
C(1C)-C(2C)	1.510(4)
C(2C)-O(2C)	1.430(3)
C(2C)-C(3C)	1.511(4)
C(3C)-O(3C)	1.426(3)
C(3C)-C(4C)	1.522(4)
C(4C)-O(4C)	1.419(3)
C(4C)-C(5C)	1.528(4)
C(5C)-O(5C)	1.429(3)
C(5C)-C(6C)	1.527(4)
C(6C)-O(6C)	1.410(4)
C(1D)-O(5D)	1.391(4)
C(1D)-O(4E)	1.413(4)
C(1D)-C(2D)	1.542(5)
C(2D)-O(2D)	1.393(5)
C(2D)-C(3D)	1.522(4)
C(3D)-O(3D)	1.422(4)
C(3D)-C(4D)	1.512(4)
O(4D)-C(4D)	1.447(3)
C(4D)-C(5D)	1.516(4)
C(5D)-O(5D)	1.433(4)
C(5D)-C(6D)	1.506(5)
C(6D)-O(6OD)	1.421(5)
C(1E)-O(5E)	1.402(3)
C(1E)-O(4F)	1.417(3)
C(1E)-C(2E)	1.522(4)
C(2E)-O(2OE)	1.415(4)
C(2E)-C(3E)	1.531(4)
C(3E)-O(3E)	1.427(4)
C(3E)-C(4E)	1.522(4)
C(4E)-O(4E)	1.423(3)
C(4E)-C(5E)	1.525(4)
C(5E)-O(5E)	1.433(3)

C(5E)-C(6E)	1.501(4)
C(6E)-O(6E)	1.434(4)
C(1F)-O(4G)	1.410(3)
C(1F)-O(5F)	1.414(3)
C(1F)-C(2F)	1.524(4)
C(2F)-O(2F)	1.417(3)
C(2F)-C(3F)	1.499(5)
C(3F)-O(3F)	1.436(3)
C(3F)-C(4F)	1.526(4)
C(4F)-O(4F)	1.427(4)
C(4F)-C(5F)	1.526(4)
C(5F)-O(5F)	1.422(4)
C(5F)-C(6F)	1.516(4)
C(6F)-O(6F)	1.420(4)
C(1G)-O(5G)	1.402(3)
C(1G)-C(2G)	1.532(4)
C(2G)-O(2G)	1.418(3)
C(2G)-C(3G)	1.509(4)
C(3G)-O(3G)	1.428(3)
C(3G)-C(4G)	1.515(3)
C(4G)-O(4G)	1.444(3)
C(4G)-C(5G)	1.533(3)
C(5G)-O(5G)	1.447(3)
C(5G)-C(6G)	1.510(4)
C(6G)-O(6G)	1.437(4)
O(5W)-O(20W)	1.10(2)
O(8W)-O(15W)#1	0.68(2)
O(8W)-O(15')#1	1.39(3)
O(9W)-O(9')	0.680(11)
O(10W)-O(10')	0.811(17)
O(11W)-O(18W)	1.43(2)
O(12W)-O(12A)	0.51(3)
O(12W)-O(12B)	0.81(3)
O(12A)-O(12B)	0.77(4)
O(13W)-O(13')	0.990(13)
O(14W)-O(19W)	1.23(2)
O(14W)-O(17W)	1.25(2)
O(14W)-O(14C)	1.722(16)
O(14B)-O(19W)	0.61(2)
O(15W)-O(8W)#2	0.68(2)
O(15W)-O(15')	0.72(2)
O(15')-O(8W)#2	1.39(3)
O(16W)-O(16')	0.99(2)
O(21W)-O(14')	1.21(5)
N(1)-C(19)	1.435(8)
N(1)-C(1)	1.488(6)
N(1)-C(20)	1.512(8)
C(1)-C(2)	1.506(7)
C(2)-C(3)	1.506(5)
C(3)-C(4)	1.340(6)
C(4)-C(18)	1.470(6)
C(4)-C(5)	1.476(5)
C(5)-C(6)	1.390(6)
C(5)-C(10)	1.394(5)
C(6)-C(7)	1.350(7)
C(7)-C(8)	1.370(10)
C(8)-C(9)	1.353(9)
C(9)-C(10)	1.394(7)
C(10)-C(11)	1.471(7)
C(11)-C(12)	1.420(8)
C(12)-C(13)	1.563(8)
C(13)-C(14)	1.379(8)

C(13)-C(18)	1.411(6)
C(14)-C(15)	1.396(10)
C(15)-C(16)	1.418(11)
C(16)-C(17)	1.300(8)
C(17)-C(18)	1.390(6)
O(5A)-C(1A)-O(4B)	110.1(2)
O(5A)-C(1A)-C(2A)	110.8(2)
O(4B)-C(1A)-C(2A)	109.4(2)
O(2A)-C(2A)-C(3A)	111.8(2)
O(2A)-C(2A)-C(1A)	108.0(2)
C(3A)-C(2A)-C(1A)	111.5(2)
O(3A)-C(3A)-C(2A)	108.3(2)
O(3A)-C(3A)-C(4A)	111.7(2)
C(2A)-C(3A)-C(4A)	110.2(2)
O(4A)-C(4A)-C(5A)	111.2(2)
O(4A)-C(4A)-C(3A)	106.7(2)
C(5A)-C(4A)-C(3A)	109.5(2)
C(1G)-O(4A)-C(4A)	117.6(2)
O(5A)-C(5A)-C(4A)	107.9(2)
O(5A)-C(5A)-C(6A)	108.2(2)
C(4A)-C(5A)-C(6A)	114.5(2)
C(1A)-O(5A)-C(5A)	113.6(2)
O(6A)-C(6A)-C(5A)	112.9(3)
O(4C)-C(1B)-O(5B)	110.6(2)
O(4C)-C(1B)-C(2B)	107.7(2)
O(5B)-C(1B)-C(2B)	109.7(2)
O(2B)-C(2B)-C(3B)	109.8(2)
O(2B)-C(2B)-C(1B)	110.1(2)
C(3B)-C(2B)-C(1B)	110.0(2)
O(3B)-C(3B)-C(2B)	109.9(2)
O(3B)-C(3B)-C(4B)	110.0(2)
C(2B)-C(3B)-C(4B)	110.0(2)
O(4B)-C(4B)-C(3B)	105.71(19)
O(4B)-C(4B)-C(5B)	111.15(19)
C(3B)-C(4B)-C(5B)	112.0(2)
C(1A)-O(4B)-C(4B)	117.42(18)
O(5B)-C(5B)-C(6B)	105.9(2)
O(5B)-C(5B)-C(4B)	109.7(2)
C(6B)-C(5B)-C(4B)	114.5(2)
C(1B)-O(5B)-C(5B)	113.86(19)
O(6B)-C(6B)-C(5B)	112.3(2)
O(4D)-C(1C)-O(5C)	111.3(2)
O(4D)-C(1C)-C(2C)	109.5(2)
O(5C)-C(1C)-C(2C)	109.4(2)
O(2C)-C(2C)-C(1C)	108.2(2)
O(2C)-C(2C)-C(3C)	110.7(2)
C(1C)-C(2C)-C(3C)	110.0(2)
O(3C)-C(3C)-C(2C)	109.9(2)
O(3C)-C(3C)-C(4C)	110.5(2)
C(2C)-C(3C)-C(4C)	111.6(2)
O(4C)-C(4C)-C(3C)	106.0(2)
O(4C)-C(4C)-C(5C)	108.7(2)
C(3C)-C(4C)-C(5C)	111.1(2)
C(1B)-O(4C)-C(4C)	119.9(2)
O(5C)-C(5C)-C(6C)	106.2(2)
O(5C)-C(5C)-C(4C)	111.6(2)
C(6C)-C(5C)-C(4C)	111.4(2)
C(1C)-O(5C)-C(5C)	114.5(2)
O(6C)-C(6C)-C(5C)	108.5(3)
O(5D)-C(1D)-O(4E)	111.8(3)
O(5D)-C(1D)-C(2D)	111.8(2)
O(4E)-C(1D)-C(2D)	107.6(3)

O(2D)-C(2D)-C(3D)	113.0(4)
O(2D)-C(2D)-C(1D)	110.7(3)
C(3D)-C(2D)-C(1D)	108.8(3)
O(3D)-C(3D)-C(4D)	111.5(2)
O(3D)-C(3D)-C(2D)	108.9(3)
C(4D)-C(3D)-C(2D)	109.0(3)
C(1C)-O(4D)-C(4D)	117.2(2)
O(4D)-C(4D)-C(3D)	107.2(2)
O(4D)-C(4D)-C(5D)	110.0(2)
C(3D)-C(4D)-C(5D)	109.9(2)
O(5D)-C(5D)-C(6D)	104.9(3)
O(5D)-C(5D)-C(4D)	109.5(3)
C(6D)-C(5D)-C(4D)	114.5(3)
C(1D)-O(5D)-C(5D)	115.0(2)
O(6OD)-C(6D)-C(5D)	110.8(3)
O(5E)-C(1E)-O(4F)	111.3(2)
O(5E)-C(1E)-C(2E)	111.1(2)
O(4F)-C(1E)-C(2E)	107.6(2)
O(2OE)-C(2E)-C(1E)	110.1(3)
O(2OE)-C(2E)-C(3E)	110.8(2)
C(1E)-C(2E)-C(3E)	110.4(2)
O(3E)-C(3E)-C(4E)	110.4(3)
O(3E)-C(3E)-C(2E)	110.0(3)
C(4E)-C(3E)-C(2E)	109.1(2)
O(4E)-C(4E)-C(3E)	107.0(2)
O(4E)-C(4E)-C(5E)	110.8(2)
C(3E)-C(4E)-C(5E)	110.9(2)
C(1D)-O(4E)-C(4E)	117.4(2)
O(5E)-C(5E)-C(6E)	107.2(2)
O(5E)-C(5E)-C(4E)	108.6(2)
C(6E)-C(5E)-C(4E)	114.7(3)
C(1E)-O(5E)-C(5E)	114.1(2)
O(6E)-C(6E)-C(5E)	111.7(3)
O(4G)-C(1F)-O(5F)	110.5(2)
O(4G)-C(1F)-C(2F)	107.7(2)
O(5F)-C(1F)-C(2F)	109.9(2)
O(2F)-C(2F)-C(3F)	111.8(2)
O(2F)-C(2F)-C(1F)	108.9(2)
C(3F)-C(2F)-C(1F)	109.7(2)
O(3F)-C(3F)-C(2F)	111.3(2)
O(3F)-C(3F)-C(4F)	108.2(2)
C(2F)-C(3F)-C(4F)	111.6(2)
O(4F)-C(4F)-C(5F)	108.9(2)
O(4F)-C(4F)-C(3F)	106.3(2)
C(5F)-C(4F)-C(3F)	111.4(2)
C(1E)-O(4F)-C(4F)	118.98(19)
O(5F)-C(5F)-C(6F)	107.1(2)
O(5F)-C(5F)-C(4F)	111.3(2)
C(6F)-C(5F)-C(4F)	113.8(2)
C(1F)-O(5F)-C(5F)	113.7(2)
O(6F)-C(6F)-C(5F)	110.0(2)
O(5G)-C(1G)-O(4A)	111.7(2)
O(5G)-C(1G)-C(2G)	109.7(2)
O(4A)-C(1G)-C(2G)	108.3(2)
O(2G)-C(2G)-C(3G)	110.2(2)
O(2G)-C(2G)-C(1G)	110.7(2)
C(3G)-C(2G)-C(1G)	110.3(2)
O(3G)-C(3G)-C(2G)	109.9(2)
O(3G)-C(3G)-C(4G)	109.7(2)
C(2G)-C(3G)-C(4G)	109.5(2)
O(4G)-C(4G)-C(3G)	106.6(2)
O(4G)-C(4G)-C(5G)	110.5(2)

C(3G)-C(4G)-C(5G)	109.8(2)
C(1F)-O(4G)-C(4G)	117.6(2)
O(5G)-C(5G)-C(6G)	105.8(2)
O(5G)-C(5G)-C(4G)	108.0(2)
C(6G)-C(5G)-C(4G)	112.4(2)
C(1G)-O(5G)-C(5G)	113.59(19)
O(6G)-C(6G)-C(5G)	107.3(2)
O(15W)#1-O(8W)-O(15')#1	10(2)
O(12A)-O(12W)-O(12B)	66(4)
O(12W)-O(12A)-O(12B)	77(5)
O(12A)-O(12B)-O(12W)	37(3)
O(19W)-O(14W)-O(17W)	127.9(14)
O(19W)-O(14W)-O(14C)	124.2(11)
O(17W)-O(14W)-O(14C)	4.2(12)
O(8W)#2-O(15W)-O(15')	161(5)
O(15W)-O(15')-O(8W)#2	9(2)
O(14B)-O(19W)-O(14W)	166(4)
C(19)-N(1)-C(1)	110.6(5)
C(19)-N(1)-C(20)	110.9(5)
C(1)-N(1)-C(20)	112.1(4)
N(1)-C(1)-C(2)	109.5(4)
C(1)-C(2)-C(3)	111.0(4)
C(4)-C(3)-C(2)	127.2(4)
C(3)-C(4)-C(18)	124.3(3)
C(3)-C(4)-C(5)	118.5(4)
C(18)-C(4)-C(5)	116.8(3)
C(6)-C(5)-C(10)	117.8(4)
C(6)-C(5)-C(4)	117.4(3)
C(10)-C(5)-C(4)	124.8(4)
C(7)-C(6)-C(5)	122.9(5)
C(6)-C(7)-C(8)	119.2(6)
C(9)-C(8)-C(7)	119.3(5)
C(8)-C(9)-C(10)	122.5(5)
C(9)-C(10)-C(5)	118.0(4)
C(9)-C(10)-C(11)	116.2(5)
C(5)-C(10)-C(11)	125.6(4)
C(12)-C(11)-C(10)	123.0(5)
C(11)-C(12)-C(13)	115.6(4)
C(14)-C(13)-C(18)	120.7(5)
C(14)-C(13)-C(12)	121.7(4)
C(18)-C(13)-C(12)	117.6(5)
C(13)-C(14)-C(15)	119.2(5)
C(14)-C(15)-C(16)	119.4(6)
C(17)-C(16)-C(15)	119.2(6)
C(16)-C(17)-C(18)	124.5(5)
C(17)-C(18)-C(13)	116.7(5)
C(17)-C(18)-C(4)	122.5(4)
C(13)-C(18)-C(4)	120.3(3)

Symmetry transformations used to generate equivalent atoms:
#1 x,y-1,z   #2 x,y+1,z

Table S11: Anisotropic displacement parameters (A^2 x 10^3) for cdamtr-16. The anisotropic displacement factor exponent takes the form: -2 pi^2 [h^2 a*^2 U11 + ... + 2 h k a* b* U12]

	U11	U22	U33	U23	U13	U12
C(1A)	36(1)	40(1)	40(2)	-1(1)	4(1)	-9(1)
C(2A)	31(1)	50(1)	40(2)	5(1)	4(1)	3(1)
O(2A)	45(1)	52(1)	60(2)	11(1)	15(1)	18(1)
C(3A)	32(1)	39(1)	43(2)	5(1)	4(1)	4(1)
O(3A)	45(1)	55(1)	62(2)	24(1)	11(1)	13(1)
C(4A)	32(1)	35(1)	36(2)	-1(1)	4(1)	-3(1)
O(4A)	31(1)	38(1)	36(1)	-2(1)	2(1)	-2(1)
C(5A)	46(1)	34(1)	40(2)	1(1)	12(1)	3(1)
O(5A)	53(1)	34(1)	51(1)	-3(1)	15(1)	-9(1)
C(6A)	73(2)	37(1)	66(2)	9(1)	25(2)	14(1)
O(6A)	99(2)	56(2)	90(2)	-26(2)	29(2)	1(2)
C(1B)	33(1)	46(1)	40(2)	0(1)	5(1)	-1(1)
C(2B)	36(1)	45(1)	48(2)	-8(1)	-1(1)	9(1)
O(2B)	68(1)	54(1)	50(2)	-19(1)	-3(1)	18(1)
C(3B)	32(1)	31(1)	45(2)	2(1)	-1(1)	3(1)
O(3B)	52(1)	31(1)	56(1)	2(1)	-3(1)	6(1)
C(4B)	28(1)	32(1)	36(2)	-2(1)	1(1)	-1(1)
O(4B)	34(1)	39(1)	37(1)	1(1)	4(1)	-6(1)
C(5B)	31(1)	33(1)	42(2)	1(1)	0(1)	2(1)
O(5B)	37(1)	42(1)	43(1)	3(1)	2(1)	-7(1)
C(6B)	52(2)	29(1)	52(2)	3(1)	-2(1)	4(1)
O(6B)	54(1)	41(1)	63(2)	0(1)	-10(1)	-10(1)
C(1C)	35(1)	40(1)	34(2)	1(1)	0(1)	-6(1)
C(2C)	38(1)	44(1)	40(2)	-5(1)	3(1)	-5(1)
O(2C)	49(1)	49(1)	65(2)	-22(1)	-1(1)	-6(1)
C(3C)	41(1)	35(1)	37(2)	-1(1)	2(1)	2(1)
O(3C)	58(1)	48(1)	64(2)	-16(1)	-7(1)	16(1)
C(4C)	36(1)	37(1)	34(2)	4(1)	1(1)	-4(1)
O(4C)	33(1)	48(1)	35(1)	2(1)	1(1)	0(1)
C(5C)	37(1)	31(1)	43(2)	-1(1)	-1(1)	-4(1)
O(5C)	40(1)	33(1)	48(1)	4(1)	-4(1)	-5(1)
C(6C)	53(2)	35(1)	69(2)	-3(1)	-14(2)	-6(1)
O(6C)	74(2)	35(1)	96(2)	-17(1)	-8(2)	4(1)
C(1D)	31(1)	85(2)	41(2)	11(2)	2(1)	-4(1)
C(2D)	42(1)	89(3)	38(2)	4(2)	0(1)	-20(2)
O(2D)	68(2)	114(2)	64(2)	-18(2)	7(1)	-50(2)
C(3D)	42(1)	57(2)	33(2)	-1(1)	-1(1)	-9(1)
O(3D)	53(1)	76(2)	44(1)	-18(1)	7(1)	-19(1)
O(4D)	34(1)	46(1)	36(1)	1(1)	2(1)	-4(1)
C(4D)	34(1)	54(2)	33(2)	-1(1)	2(1)	-2(1)
C(5D)	40(1)	59(2)	41(2)	-1(1)	8(1)	0(1)
O(5D)	40(1)	70(2)	58(2)	7(1)	13(1)	5(1)
C(6D)	51(2)	58(2)	78(3)	-15(2)	12(2)	3(2)
O(6OD)	68(2)	69(2)	100(2)	14(2)	13(2)	2(1)
C(1E)	30(1)	48(1)	40(2)	8(1)	2(1)	4(1)
C(2E)	39(1)	49(2)	45(2)	3(1)	-3(1)	0(1)
O(2OE)	71(2)	49(1)	74(2)	12(1)	4(1)	0(1)
C(3E)	45(1)	51(2)	38(2)	-1(1)	5(1)	7(1)
O(3E)	91(2)	63(2)	66(2)	-15(1)	19(2)	5(1)
C(4E)	32(1)	51(2)	36(2)	6(1)	5(1)	1(1)
O(4E)	32(1)	85(2)	34(1)	9(1)	4(1)	3(1)
C(5E)	33(1)	55(2)	32(2)	5(1)	3(1)	-1(1)
O(5E)	35(1)	50(1)	38(1)	9(1)	6(1)	9(1)
C(6E)	51(2)	52(2)	45(2)	5(1)	4(1)	-2(1)

O(6E)	52(1)	61(1)	62(2)	22(1)	2(1)	10(1)
C(1F)	32(1)	42(1)	31(2)	5(1)	2(1)	2(1)
C(2F)	35(1)	39(1)	39(2)	10(1)	2(1)	-2(1)
O(2F)	47(1)	43(1)	62(2)	22(1)	-12(1)	-6(1)
C(3F)	36(1)	35(1)	43(2)	1(1)	1(1)	6(1)
O(3F)	52(1)	36(1)	66(2)	-6(1)	-15(1)	4(1)
C(4F)	31(1)	41(1)	31(2)	3(1)	4(1)	6(1)
O(4F)	30(1)	58(1)	33(1)	6(1)	3(1)	8(1)
C(5F)	39(1)	37(1)	41(2)	6(1)	4(1)	2(1)
O(5F)	39(1)	38(1)	42(1)	1(1)	1(1)	9(1)
C(6F)	56(2)	36(1)	53(2)	8(1)	-3(1)	7(1)
O(6F)	54(1)	55(1)	66(2)	10(1)	-1(1)	16(1)
C(1G)	31(1)	41(1)	37(2)	-1(1)	-1(1)	-3(1)
C(2G)	37(1)	44(1)	36(2)	7(1)	0(1)	-1(1)
O(2G)	36(1)	54(1)	70(2)	23(1)	-4(1)	4(1)
C(3G)	36(1)	30(1)	40(2)	7(1)	0(1)	3(1)
O(3G)	38(1)	36(1)	74(2)	21(1)	-5(1)	0(1)
C(4G)	29(1)	31(1)	39(2)	-1(1)	-1(1)	-1(1)
O(4G)	30(1)	38(1)	38(1)	1(1)	0(1)	1(1)
C(5G)	35(1)	30(1)	48(2)	3(1)	1(1)	-1(1)
O(5G)	37(1)	36(1)	51(1)	-9(1)	4(1)	-1(1)
C(6G)	40(1)	31(1)	116(3)	8(2)	7(2)	3(1)
O(6G)	60(1)	35(1)	130(3)	21(1)	-1(2)	2(1)
O(1W)	80(2)	59(2)	118(3)	9(2)	-17(2)	-8(1)
O(2W)	87(2)	51(1)	55(2)	6(1)	3(1)	12(1)
O(3W)	81(2)	84(2)	86(2)	-14(2)	-15(2)	17(2)
O(4W)	91(2)	49(2)	149(3)	-19(2)	-20(2)	-11(2)
O(5W)	71(2)	70(2)	70(3)	21(2)	13(2)	14(2)
O(6W)	84(3)	86(3)	72(3)	5(3)	-2(3)	5(3)
O(7W)	99(6)	105(6)	60(5)	16(4)	27(4)	-16(5)
O(8W)	64(6)	120(12)	150(14)	-71(11)	-16(7)	8(7)
O(9W)	58(5)	81(8)	47(6)	-19(6)	6(4)	7(6)
O(9')	74(3)	47(2)	69(3)	0(2)	-2(2)	-5(2)
O(10W)	47(2)	71(2)	101(5)	6(3)	10(2)	31(2)
O(11W)	111(4)	149(6)	140(6)	14(5)	58(4)	10(4)
O(12W)	184(13)	60(5)	48(8)	-2(4)	-9(8)	-26(6)
O(13W)	80(4)	125(5)	39(3)	-32(3)	4(3)	-19(4)
O(14W)	48(2)	118(4)	65(3)	-20(2)	16(2)	3(2)
N(1)	138(4)	47(2)	60(2)	4(2)	7(2)	8(2)
C(1)	105(3)	56(2)	65(3)	13(2)	24(2)	-1(2)
C(2)	87(3)	54(2)	85(3)	-10(2)	36(2)	-2(2)
C(3)	85(3)	46(2)	90(3)	9(2)	41(2)	4(2)
C(4)	54(2)	50(2)	72(3)	3(2)	21(2)	-11(1)
C(5)	55(2)	60(2)	64(2)	-2(2)	11(2)	-5(2)
C(6)	74(3)	62(2)	137(5)	11(3)	26(3)	-8(2)
C(7)	124(5)	53(3)	229(9)	-19(4)	61(6)	-7(3)
C(8)	189(8)	54(3)	184(7)	-16(4)	63(7)	12(4)
C(9)	109(4)	98(4)	104(4)	-6(3)	34(3)	39(3)
C(10)	56(2)	88(3)	71(3)	11(2)	15(2)	4(2)
C(11)	59(2)	118(4)	149(5)	50(4)	28(3)	5(3)
C(12)	66(3)	73(3)	217(7)	-22(4)	69(4)	-30(2)
C(13)	82(3)	40(2)	95(3)	3(2)	29(2)	-2(2)
C(14)	117(4)	53(2)	100(4)	-8(2)	33(3)	-11(2)
C(15)	190(8)	48(2)	130(5)	-6(3)	-48(5)	-7(4)
C(16)	91(3)	55(2)	140(5)	-9(3)	-16(3)	-11(2)
C(17)	67(2)	52(2)	107(4)	-4(2)	7(2)	-7(2)
C(18)	49(2)	41(2)	100(3)	-17(2)	16(2)	-7(1)
C(19)	220(8)	73(3)	90(4)	-8(3)	-32(5)	6(4)
C(20)	150(6)	88(4)	119(5)	14(3)	54(4)	41(4)

Table S12: Hydrogen coordinates (x 10^4) and isotropic displacement parameters (A^2 x 10^3) for cdamtr-16.

	x	y	z	U(eq)
H(1A)	3099	1173	7702	46
H(2A)	2933	504	8353	48
H(2OA)	2973	-591	8011	78
H(3A)	1351	-96	8011	46
H(3OA)	1332	-481	8734	81
H(4A)	1464	970	8703	41
H(5A)	841	1116	7800	48
H(6A1)	671	2357	7960	70
H(6A2)	-17	1878	8217	70
H(6OA)	743	2024	8819	122
H(1B)	2839	1009	5687	48
H(2B)	3539	306	6248	52
H(2OB)	3084	-395	5743	86
H(3B)	1851	100	6636	43
H(3OB)	2805	-368	7175	69
H(4B)	3248	904	7026	38
H(5B)	1683	1509	6655	43
H(6B1)	2377	2630	6735	53
H(6B2)	2232	2268	7209	53
H(6OB)	3540	2365	7288	79
H(1C)	-834	1125	4801	43
H(2C)	534	590	4684	49
H(2OC)	43	-506	4759	82
H(3C)	461	136	5600	45
H(3OC)	1839	-287	5445	85
H(4C)	1705	1159	5276	43
H(5C)	227	1532	5825	44
H(6C1)	1471	2338	5836	63
H(6C2)	1321	2481	5316	63
H(6OC)	167	3262	5875	103
H(1D)	-4274	919	5735	63
H(2D)	-3501	569	5088	68
H(2OD)	-3710	-546	5613	123
H(3D)	-2219	-39	5674	53
H(3OD)	-1562	-219	5003	86
H(4D)	-2053	1195	5108	48
H(5D)	-2081	1143	6073	55
H(6D1)	-2094	2383	6077	75
H(6D2)	-1261	2141	5801	75
H(6OD)	-1868	2482	5206	119
H(1E)	-5552	-200	7479	47
H(2E)	-5478	-692	6757	53
H(2E)	-5291	-1519	7223	97
H(3E)	-3614	-532	6786	54
H(3OE)	-4054	-787	6013	110
H(4E)	-4804	286	6245	48
H(5E)	-3842	780	7014	48
H(6E1)	-4616	1861	6950	59
H(6E2)	-4018	1739	6523	59
H(6OE)	-5694	1812	6537	87
H(1F)	-3320	39	9208	42
H(2F)	-4386	-739	8896	45
H(2OF)	-3587	-1564	9069	76
H(3F)	-3323	-791	8113	45
H(3OF)	-4054	-1737	8094	77

H(4F)	-5014	-132	8135	41
H(5F)	-3401	646	8105	47
H(6F1)	-4232	1682	8239	58
H(6F2)	-4558	1307	7792	58
H(6OF)	-5349	1112	8575	88
H(1G)	508	837	9181	44
H(2G)	-485	44	9523	47
H(2OG)	826	-389	9173	80
H(3G)	-964	-431	8644	42
H(3OG)	-1095	-1236	9200	74
H(4G)	-2017	388	9256	40
H(5G)	-1248	875	8421	45
H(6G1)	-2564	1506	8575	75
H(6G2)	-2228	1686	9067	75
H(6OG)	-1922	2558	8562	113
H(11)	-646	-1664	5890	90
H(12)	-18	-1177	5597	90
H(21)	1001	-1141	6192	90
H(22)	392	-1643	6484	90
H(3)	-260	-277	6220	89
H(6)	198	791	6619	109
H(7)	-249	1916	6808	162
H(8)	-1548	2063	7207	171
H(9)	-2208	1086	7521	124
H(11A)	-1953	-137	7788	130
H(11B)	-2536	-237	7360	130
H(12A)	-1795	-1170	7141	143
H(12B)	-2052	-1271	7650	143
H(14)	-891	-1629	8172	108
H(15)	596	-1846	8321	147
H(16)	1653	-1567	7763	114
H(17)	1253	-963	7153	91
H(19A)	-640	-2707	5479	192
H(19B)	220	-3022	5261	192
H(19C)	-172	-2295	5083	192
H(20A)	1624	-2454	5381	179
H(20B)	1686	-1785	5704	179
H(20C)	1268	-1703	5221	179

Table S13: Torsion angles [deg] for cdamtr-16.

O(5A)-C(1A)-C(2A)-O(2A)	174.5(2)
O(4B)-C(1A)-C(2A)-O(2A)	53.0(3)
O(5A)-C(1A)-C(2A)-C(3A)	51.3(3)
O(4B)-C(1A)-C(2A)-C(3A)	-70.2(3)
O(2A)-C(2A)-C(3A)-O(3A)	66.4(3)
C(1A)-C(2A)-C(3A)-O(3A)	-172.5(2)
O(2A)-C(2A)-C(3A)-C(4A)	-171.2(2)
C(1A)-C(2A)-C(3A)-C(4A)	-50.1(3)
O(3A)-C(3A)-C(4A)-O(4A)	-63.7(3)
C(2A)-C(3A)-C(4A)-O(4A)	175.9(2)
O(3A)-C(3A)-C(4A)-C(5A)	175.8(2)
C(2A)-C(3A)-C(4A)-C(5A)	55.5(3)
C(5A)-C(4A)-O(4A)-C(1G)	-114.9(3)
C(3A)-C(4A)-O(4A)-C(1G)	125.7(2)
O(4A)-C(4A)-C(5A)-O(5A)	-178.3(2)
C(3A)-C(4A)-C(5A)-O(5A)	-60.7(3)
O(4A)-C(4A)-C(5A)-C(6A)	61.1(3)
C(3A)-C(4A)-C(5A)-C(6A)	178.7(3)
O(4B)-C(1A)-O(5A)-C(5A)	61.7(3)
C(2A)-C(1A)-O(5A)-C(5A)	-59.4(3)
C(4A)-C(5A)-O(5A)-C(1A)	64.4(3)
C(6A)-C(5A)-O(5A)-C(1A)	-171.1(3)
O(5A)-C(5A)-C(6A)-O(6A)	-69.6(3)
C(4A)-C(5A)-C(6A)-O(6A)	50.7(4)
O(4C)-C(1B)-C(2B)-O(2B)	58.6(3)
O(5B)-C(1B)-C(2B)-O(2B)	179.0(2)
O(4C)-C(1B)-C(2B)-C(3B)	-62.4(3)
O(5B)-C(1B)-C(2B)-C(3B)	58.0(3)
O(2B)-C(2B)-C(3B)-O(3B)	63.6(3)
C(1B)-C(2B)-C(3B)-O(3B)	-175.2(2)
O(2B)-C(2B)-C(3B)-C(4B)	-175.2(2)
C(1B)-C(2B)-C(3B)-C(4B)	-54.0(3)
O(3B)-C(3B)-C(4B)-O(4B)	-65.2(2)
C(2B)-C(3B)-C(4B)-O(4B)	173.63(19)
O(3B)-C(3B)-C(4B)-C(5B)	173.6(2)
C(2B)-C(3B)-C(4B)-C(5B)	52.4(3)
O(5A)-C(1A)-O(4B)-C(4B)	116.2(2)
C(2A)-C(1A)-O(4B)-C(4B)	-121.8(2)
C(3B)-C(4B)-O(4B)-C(1A)	139.7(2)
C(5B)-C(4B)-O(4B)-C(1A)	-98.6(2)
O(4B)-C(4B)-C(5B)-O(5B)	-171.17(18)
C(3B)-C(4B)-C(5B)-O(5B)	-53.2(3)
O(4B)-C(4B)-C(5B)-C(6B)	69.9(3)
C(3B)-C(4B)-C(5B)-C(6B)	-172.1(2)
O(4C)-C(1B)-O(5B)-C(5B)	57.3(3)
C(2B)-C(1B)-O(5B)-C(5B)	-61.4(3)
C(6B)-C(5B)-O(5B)-C(1B)	-177.2(2)
C(4B)-C(5B)-O(5B)-C(1B)	58.7(3)
O(5B)-C(5B)-C(6B)-O(6B)	-65.9(3)
C(4B)-C(5B)-C(6B)-O(6B)	55.1(3)
O(4D)-C(1C)-C(2C)-O(2C)	57.6(3)
O(5C)-C(1C)-C(2C)-O(2C)	179.7(2)
O(4D)-C(1C)-C(2C)-C(3C)	-63.5(3)
O(5C)-C(1C)-C(2C)-C(3C)	58.7(3)
O(2C)-C(2C)-C(3C)-O(3C)	63.8(3)
C(1C)-C(2C)-C(3C)-O(3C)	-176.6(2)
O(2C)-C(2C)-C(3C)-C(4C)	-173.3(2)
C(1C)-C(2C)-C(3C)-C(4C)	-53.7(3)
O(3C)-C(3C)-C(4C)-O(4C)	-70.8(3)

C(2C)-C(3C)-C(4C)-O(4C)	166.6(2)
O(3C)-C(3C)-C(4C)-C(5C)	171.2(2)
C(2C)-C(3C)-C(4C)-C(5C)	48.7(3)
O(5B)-C(1B)-O(4C)-C(4C)	108.7(3)
C(2B)-C(1B)-O(4C)-C(4C)	-131.4(2)
C(3C)-C(4C)-O(4C)-C(1B)	121.0(2)
C(5C)-C(4C)-O(4C)-C(1B)	-119.5(2)
O(4C)-C(4C)-C(5C)-O(5C)	-165.0(2)
C(3C)-C(4C)-C(5C)-O(5C)	-48.7(3)
O(4C)-C(4C)-C(5C)-C(6C)	76.5(3)
C(3C)-C(4C)-C(5C)-C(6C)	-167.3(3)
O(4D)-C(1C)-O(5C)-C(5C)	59.5(3)
C(2C)-C(1C)-O(5C)-C(5C)	-61.6(3)
C(6C)-C(5C)-O(5C)-C(1C)	178.5(2)
C(4C)-C(5C)-O(5C)-C(1C)	56.9(3)
O(5C)-C(5C)-C(6C)-O(6C)	65.1(3)
C(4C)-C(5C)-C(6C)-O(6C)	-173.1(3)
O(5D)-C(1D)-C(2D)-O(2D)	179.4(3)
O(4E)-C(1D)-C(2D)-O(2D)	56.4(3)
O(5D)-C(1D)-C(2D)-C(3D)	54.7(4)
O(4E)-C(1D)-C(2D)-C(3D)	-68.4(3)
O(2D)-C(2D)-C(3D)-O(3D)	59.2(4)
C(1D)-C(2D)-C(3D)-O(3D)	-177.4(3)
O(2D)-C(2D)-C(3D)-C(4D)	-178.9(3)
C(1D)-C(2D)-C(3D)-C(4D)	-55.5(4)
O(5C)-C(1C)-O(4D)-C(4D)	104.4(3)
C(2C)-C(1C)-O(4D)-C(4D)	-134.5(2)
C(1C)-O(4D)-C(4D)-C(3D)	116.8(3)
C(1C)-O(4D)-C(4D)-C(5D)	-123.7(3)
O(3D)-C(3D)-C(4D)-O(4D)	-61.9(3)
C(2D)-C(3D)-C(4D)-O(4D)	177.8(2)
O(3D)-C(3D)-C(4D)-C(5D)	178.6(2)
C(2D)-C(3D)-C(4D)-C(5D)	58.3(3)
O(4D)-C(4D)-C(5D)-O(5D)	-175.3(2)
C(3D)-C(4D)-C(5D)-O(5D)	-57.6(3)
O(4D)-C(4D)-C(5D)-C(6D)	67.2(4)
C(3D)-C(4D)-C(5D)-C(6D)	-175.1(3)
O(4E)-C(1D)-O(5D)-C(5D)	63.5(3)
C(2D)-C(1D)-O(5D)-C(5D)	-57.2(4)
C(6D)-C(5D)-O(5D)-C(1D)	-178.5(3)
C(4D)-C(5D)-O(5D)-C(1D)	58.1(4)
O(5D)-C(5D)-C(6D)-O(6OD)	-56.4(3)
C(4D)-C(5D)-C(6D)-O(6OD)	63.7(4)
O(5E)-C(1E)-C(2E)-O(2OE)	177.6(2)
O(4F)-C(1E)-C(2E)-O(2OE)	55.6(3)
O(5E)-C(1E)-C(2E)-C(3E)	54.9(3)
O(4F)-C(1E)-C(2E)-C(3E)	-67.1(3)
O(2OE)-C(2E)-C(3E)-O(3E)	64.1(3)
C(1E)-C(2E)-C(3E)-O(3E)	-173.6(3)
O(2OE)-C(2E)-C(3E)-C(4E)	-174.7(3)
C(1E)-C(2E)-C(3E)-C(4E)	-52.4(3)
O(3E)-C(3E)-C(4E)-O(4E)	-63.6(3)
C(2E)-C(3E)-C(4E)-O(4E)	175.4(2)
O(3E)-C(3E)-C(4E)-C(5E)	175.6(2)
C(2E)-C(3E)-C(4E)-C(5E)	54.6(3)
O(5D)-C(1D)-O(4E)-C(4E)	120.4(3)
C(2D)-C(1D)-O(4E)-C(4E)	-116.6(3)
C(3E)-C(4E)-O(4E)-C(1D)	140.1(3)
C(5E)-C(4E)-O(4E)-C(1D)	-99.0(3)
O(4E)-C(4E)-C(5E)-O(5E)	-176.0(2)
C(3E)-C(4E)-C(5E)-O(5E)	-57.4(3)
O(4E)-C(4E)-C(5E)-C(6E)	64.1(3)

C(3E)-C(4E)-C(5E)-C(6E)	-177.3(2)
O(4F)-C(1E)-O(5E)-C(5E)	59.4(3)
C(2E)-C(1E)-O(5E)-C(5E)	-60.5(3)
C(6E)-C(5E)-O(5E)-C(1E)	-174.5(2)
C(4E)-C(5E)-O(5E)-C(1E)	61.1(3)
O(5E)-C(5E)-C(6E)-O(6E)	-64.6(3)
C(4E)-C(5E)-C(6E)-O(6E)	56.0(3)
O(4G)-C(1F)-C(2F)-O(2F)	60.8(3)
O(5F)-C(1F)-C(2F)-O(2F)	-178.8(2)
O(4G)-C(1F)-C(2F)-C(3F)	-61.9(3)
O(5F)-C(1F)-C(2F)-C(3F)	58.5(3)
O(2F)-C(2F)-C(3F)-O(3F)	65.5(3)
C(1F)-C(2F)-C(3F)-O(3F)	-173.61(19)
O(2F)-C(2F)-C(3F)-C(4F)	-173.5(2)
C(1F)-C(2F)-C(3F)-C(4F)	-52.6(3)
O(3F)-C(3F)-C(4F)-O(4F)	-69.9(3)
C(2F)-C(3F)-C(4F)-O(4F)	167.3(2)
O(3F)-C(3F)-C(4F)-C(5F)	171.5(2)
C(2F)-C(3F)-C(4F)-C(5F)	48.7(3)
O(5E)-C(1E)-O(4F)-C(4F)	103.5(3)
C(2E)-C(1E)-O(4F)-C(4F)	-134.6(2)
C(5F)-C(4F)-O(4F)-C(1E)	-112.9(2)
C(3F)-C(4F)-O(4F)-C(1E)	127.0(2)
O(4F)-C(4F)-C(5F)-O(5F)	-166.79(19)
C(3F)-C(4F)-C(5F)-O(5F)	-49.8(3)
O(4F)-C(4F)-C(5F)-C(6F)	72.1(3)
C(3F)-C(4F)-C(5F)-C(6F)	-170.9(2)
O(4G)-C(1F)-O(5F)-C(5F)	56.2(3)
C(2F)-C(1F)-O(5F)-C(5F)	-62.5(3)
C(6F)-C(5F)-O(5F)-C(1F)	-176.8(2)
C(4F)-C(5F)-O(5F)-C(1F)	58.2(3)
O(5F)-C(5F)-C(6F)-O(6F)	-63.7(3)
C(4F)-C(5F)-C(6F)-O(6F)	59.8(3)
C(4A)-O(4A)-C(1G)-O(5G)	108.9(2)
C(4A)-O(4A)-C(1G)-C(2G)	-130.1(2)
O(5G)-C(1G)-C(2G)-O(2G)	178.6(2)
O(4A)-C(1G)-C(2G)-O(2G)	56.3(3)
O(5G)-C(1G)-C(2G)-C(3G)	56.4(3)
O(4A)-C(1G)-C(2G)-C(3G)	-65.9(3)
O(2G)-C(2G)-C(3G)-O(3G)	62.4(3)
C(1G)-C(2G)-C(3G)-O(3G)	-175.1(2)
O(2G)-C(2G)-C(3G)-C(4G)	-177.1(2)
C(1G)-C(2G)-C(3G)-C(4G)	-54.6(3)
O(3G)-C(3G)-C(4G)-O(4G)	-63.3(3)
C(2G)-C(3G)-C(4G)-O(4G)	176.0(2)
O(3G)-C(3G)-C(4G)-C(5G)	177.0(2)
C(2G)-C(3G)-C(4G)-C(5G)	56.3(3)
O(5F)-C(1F)-O(4G)-C(4G)	103.8(2)
C(2F)-C(1F)-O(4G)-C(4G)	-136.3(2)
C(3G)-C(4G)-O(4G)-C(1F)	123.3(2)
C(5G)-C(4G)-O(4G)-C(1F)	-117.5(2)
O(4G)-C(4G)-C(5G)-O(5G)	-175.60(19)
C(3G)-C(4G)-C(5G)-O(5G)	-58.3(3)
O(4G)-C(4G)-C(5G)-C(6G)	68.1(3)
C(3G)-C(4G)-C(5G)-C(6G)	-174.7(3)
O(4A)-C(1G)-O(5G)-C(5G)	58.6(3)
C(2G)-C(1G)-O(5G)-C(5G)	-61.6(3)
C(6G)-C(5G)-O(5G)-C(1G)	-176.8(2)
C(4G)-C(5G)-O(5G)-C(1G)	62.6(3)
O(5G)-C(5G)-C(6G)-O(6G)	57.8(4)
C(4G)-C(5G)-C(6G)-O(6G)	175.4(3)
O(17W)-O(14W)-O(19W)-O(14B)	-15(16)

O(14C)-O(14W)-O(19W)-O(14B)	-18(16)
C(19)-N(1)-C(1)-C(2)	164.0(4)
C(20)-N(1)-C(1)-C(2)	-71.7(5)
N(1)-C(1)-C(2)-C(3)	-178.5(3)
C(1)-C(2)-C(3)-C(4)	137.4(5)
C(2)-C(3)-C(4)-C(18)	1.5(7)
C(2)-C(3)-C(4)-C(5)	174.9(4)
C(3)-C(4)-C(5)-C(6)	-50.5(6)
C(18)-C(4)-C(5)-C(6)	123.4(4)
C(3)-C(4)-C(5)-C(10)	131.1(5)
C(18)-C(4)-C(5)-C(10)	-55.0(5)
C(10)-C(5)-C(6)-C(7)	2.9(10)
C(4)-C(5)-C(6)-C(7)	-175.6(7)
C(5)-C(6)-C(7)-C(8)	-4.8(13)
C(6)-C(7)-C(8)-C(9)	5.7(14)
C(7)-C(8)-C(9)-C(10)	-5.0(13)
C(8)-C(9)-C(10)-C(5)	3.1(10)
C(8)-C(9)-C(10)-C(11)	177.7(7)
C(6)-C(5)-C(10)-C(9)	-1.9(7)
C(4)-C(5)-C(10)-C(9)	176.4(5)
C(6)-C(5)-C(10)-C(11)	-175.9(6)
C(4)-C(5)-C(10)-C(11)	2.5(8)
C(9)-C(10)-C(11)-C(12)	-173.7(7)
C(5)-C(10)-C(11)-C(12)	0.3(10)
C(10)-C(11)-C(12)-C(13)	51.3(10)
C(11)-C(12)-C(13)-C(14)	108.0(6)
C(11)-C(12)-C(13)-C(18)	-70.9(7)
C(18)-C(13)-C(14)-C(15)	-1.9(7)
C(12)-C(13)-C(14)-C(15)	179.3(5)
C(13)-C(14)-C(15)-C(16)	-2.1(7)
C(14)-C(15)-C(16)-C(17)	4.8(8)
C(15)-C(16)-C(17)-C(18)	-3.5(7)
C(16)-C(17)-C(18)-C(13)	-0.5(6)
C(16)-C(17)-C(18)-C(4)	172.4(4)
C(14)-C(13)-C(18)-C(17)	3.2(6)
C(12)-C(13)-C(18)-C(17)	-177.9(4)
C(14)-C(13)-C(18)-C(4)	-169.9(4)
C(12)-C(13)-C(18)-C(4)	9.1(6)
C(3)-C(4)-C(18)-C(17)	59.3(6)
C(5)-C(4)-C(18)-C(17)	-114.2(4)
C(3)-C(4)-C(18)-C(13)	-128.0(4)
C(5)-C(4)-C(18)-C(13)	58.5(5)

Symmetry transformations used to generate equivalent atoms:
#1 x,y-1,z   #2 x,y+1,z

Table S14: Hydrogen bonds for cdamtr-16 [A and deg.].

D-H...A	d(D-H)	d(H...A)	d(D...A)	<(DHA)
O(3A)-H(3OA)...O(2G)	0.82	2.04	2.840(3)	164.7
O(6A)-H(6OA)...O(5G)	0.82	2.71	3.335(4)	134.8
O(6A)-H(6OA)...O(4A)	0.82	2.98	3.423(3)	116.3
O(3B)-H(3OB)...O(2A)	0.82	2.08	2.876(3)	164.8
O(3B)-H(3OB)...O(4B)	0.82	2.39	2.797(3)	111.6
O(3C)-H(3OC)...O(2B)	0.89	1.97	2.850(4)	173.5
O(6C)-H(6OC)...O(2G)#3	0.94	2.29	3.082(3)	141.7
O(2D)-H(2OD)...O(3E)	0.82	2.17	2.899(4)	148.9
O(3D)-H(3OD)...O(2C)	0.82	1.98	2.788(3)	169.3
O(3E)-H(3OE)...O(2D)	0.82	2.08	2.899(4)	175.0
O(6E)-H(6OE)...O(6B)#4	0.82	2.10	2.909(4)	167.5
O(3F)-H(3OF)...O(6B)#5	0.82	2.04	2.848(3)	166.7
O(3G)-H(3OG)...O(6C)#5	0.82	1.99	2.796(3)	167.0
C(1)-H(11)...O(6A)#5	0.97	2.45	2.997(5)	115.1
C(1E)-H(1E)...O(2A)#4	0.98	2.44	3.407(3)	168.0

Symmetry transformations used to generate equivalent atoms:
#1 x,y-1,z #2 x,y+1,z #3 -x,y+1/2,-z+3/2
#4 x-1,y,z #5 -x,y-1/2,-z+3/2

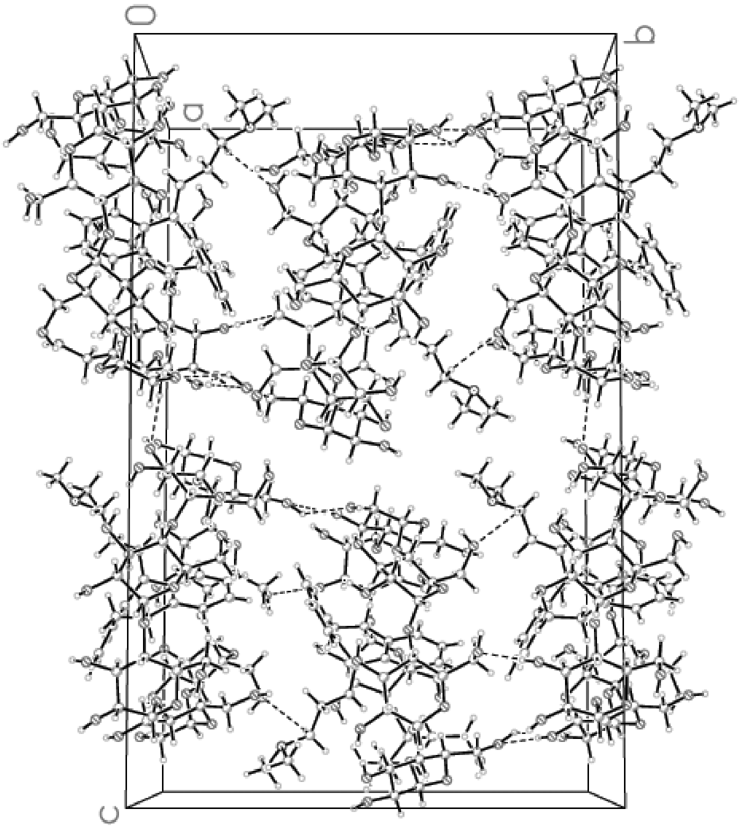

Figure S4: Packing diagram of complex **2**.
